# p53 isoforms differentially impact on the POLι dependent DNA damage tolerance pathway

**DOI:** 10.1038/s41419-021-04224-3

**Published:** 2021-10-13

**Authors:** Yitian Guo, Melanie Rall-Scharpf, Jean-Christophe Bourdon, Lisa Wiesmüller, Stephanie Biber

**Affiliations:** 1 Department of Obstetrics and Gynecology, Ulm University, Ulm, 89075 Germany; 2 Jacqui Wood Cancer Centre, School of Medicine, University of Dundee, Dundee, UK

**Keywords:** Mechanisms of disease, Tumour-suppressor proteins

## Abstract

The recently discovered p53-dependent DNA damage tolerance (DDT) pathway relies on its biochemical activities in DNA-binding, oligomerization, as well as complex formation with the translesion synthesis (TLS) polymerase iota (POLι). These p53-POLι complexes slow down nascent DNA synthesis for safe, homology-directed bypass of DNA replication barriers. In this study, we demonstrate that the alternative p53-isoforms p53β, p53γ, Δ40p53α, Δ133p53α, and Δ160p53α differentially affect this p53-POLι-dependent DDT pathway originally described for canonical p53α. We show that the C-terminal isoforms p53β and p53γ, comprising a truncated oligomerization domain (OD), bind PCNA. Conversely, N-terminally truncated isoforms have a reduced capacity to engage in this interaction. Regardless of the specific loss of biochemical activities required for this DDT pathway, all alternative isoforms were impaired in promoting POLι recruitment to PCNA in the chromatin and in decelerating DNA replication under conditions of enforced replication stress after Mitomycin C (MMC) treatment. Consistent with this, all alternative p53-isoforms no longer stimulated recombination, i.e., bypass of endogenous replication barriers. Different from the other isoforms, Δ133p53α and Δ160p53α caused a severe DNA replication problem, namely fork stalling even in untreated cells. Co-expression of each alternative p53-isoform together with p53α exacerbated the DDT pathway defects, unveiling impaired POLι recruitment and replication deceleration already under unperturbed conditions. Such an inhibitory effect on p53α was particularly pronounced in cells co-expressing Δ133p53α or Δ160p53α. Notably, this effect became evident after the expression of the isoforms in tumor cells, as well as after the knockdown of endogenous isoforms in human hematopoietic stem and progenitor cells. In summary, mimicking the situation found to be associated with many cancer types and stem cells, i.e., co-expression of alternative p53-isoforms with p53α, carved out interference with p53α functions in the p53-POLι-dependent DDT pathway.

## Introduction

**Fig. 1:**
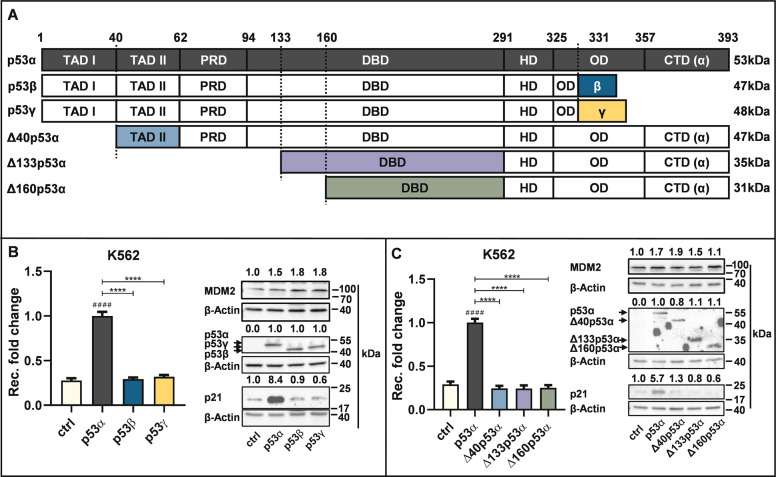
Analysis of the p53 isoforms p53α, p53β, p53γ, Δ40p53α, Δ133p53α, and Δ160p53α in replication-associated recombination. K562 (*HR-EGFP/3*′*-EGFP*) cells were transfected with 10 µg expression plasmid for p53α and corresponding amounts for p53β, p53γ, Δ40p53α, Δ133p53α, Δ160p53α or empty vector (EV) in controls (ctrl) as indicated in the graphs. Seventy-two hours after transfection FACS analysis (left panels of **B**, **C**) and protein harvesting (right panels of **B**, **C**) were performed. Recombination (rec.) fold changes were analyzed flow cytometrically by quantification of EGFP-positive cells among living cells. Mean values from p53α-expressing cells were set to 1 (absolute mean frequencies: 4*10^−5^). Data were collected from ≥18 individual measurements each. For graphic presentation, calculation of SEM and statistically significant differences via Kruskal-Wallis test followed by two-tailed Mann−Whitney U test GraphPadPrism8.4 software was used. # indicates a statistically significant difference between ctrl and the respective p53-isoform data. ****(# # # #) *P* < 0.0001. Quantification of protein levels was carried out using ImageLab software, normalized to β-actin, and indicated above the representative immunoblot. **A** Schematic overview of different domains of p53 isoforms. p53α (highlighted in black) consists of transactivation domain I (TAD I), transactivation domain II (TAD II), proline-rich domain (PRD), DNA-binding domain, hinge domain (HD), oligomerization domain (OD), C-terminal domain (CTD) while parts of the other p53-isoforms are replaced by other sequences or missing. Note that the color code in the scheme was used in the subsequent graphs. **B** Role of p53β and p53γ in replication-associated recombination. Left panel shows Recombination (Rec.) fold changes. Right panel shows representative Western Blot analysis of the indicated p53 isoforms. β-Actin served as a loading control. **C** Role of p53α, Δ40p53α, Δ133p53α, and Δ160p53α in replication-associated recombination. Left panel shows Rec. fold changes. Right panel shows representative Western Blot analysis of the indicated p53 isoforms. β-Actin served as loading control.

Already in the nineties, shortly after p53 was recognized to be a tumor suppressor [1, 2], it became clear that it is a key player in the protection of the genome integrity [3]. Simultaneously, with the discovery of p53′s canonical functions in transcriptionally activating cell cycle-regulatory and pro-apoptotic genes through sequence-specific DNA-binding [4–7], non-canonical functions of p53 in DNA repair, recombination, and replication, not requiring its transcriptional activity, emerged [8–14]. After more than 25 years of research, it came as a surprise when twelve *TP53*-isoforms were identified, which are generated by the use of different promoters, alternative splicing, and the internal ribosome entry site [15, 16]. *TP53* gene products other than the canonical p53α lack N- and C-terminal domains of the human protein with well-defined biochemical functions like the first transcriptional transactivation domain (TAD1, amino acids [aa] 1 to 39 in p53α) in Δ40p53α (Fig. 1A). These alternative p53-isoforms were then found to be differentially expressed in normal and cancer tissues, revealing pro-survival features of N-terminally truncated Δ133p53 and Δ160p53 in a p53α-dependent and -independent manner [15]. Therefore, co-expression of alternative p53-isoforms provides an additional mechanism to modulate p53α´s tumor suppressor functions beyond *TP53* gene mutations mostly affecting the DNA-binding domain (DBD) [17].

While the canonical activities of p53-isoforms have been investigated quite intensively [18], non-canonical functions in stabilizing the genome have remained largely obscure. Recently, we discovered that p53α, via interactions with proliferating cell nuclear antigen (PCNA) and the translesion synthesis (TLS) polymerase iota (POLι), regulates a homology-directed DDT-pathway [19, 20]. This p53-POLι-dependent DDT-pathway serves to bypass barriers during DNA replication and may confer a pro-survival effect to stem cells from which, however, cancer stem cells also benefit [19–22]. In this study, we analyzed the role of the p53-isoforms p53β, p53γ, Δ40p53α, Δ133p53α, and Δ160p53α in this p53-POLι-dependent DDT through the analyses of replication-associated recombination, DNA replication dynamics, and key protein interactions. We provide evidence that these alternative p53-isoforms differentially lost biochemical functions in the homology-directed resolution of replication barriers. When co-expressed with p53α, thereby mimicking their status in cancer tissues, alternative p53-isoforms block the p53-POLι-dependent DDT-pathway which unlocks faster and likely more mutagenic bypass mechanisms.

## Results

### The p53-isoforms p53β, p53γ, Δ40p53α, Δ133p53α, and Δ160p53α have lost p53α′s activity to stimulate replication-associated recombination

Our previous work demonstrated that p53α bypasses replication obstacles via a homology-directed DDT-pathway which can be detected by measurements of spontaneous, i.e., replication-associated recombination events [19]. To investigate the role of N-terminally truncated p53-isoforms (ΔN) Δ40p53α, Δ133p53α, and Δ160p53α, as well as C-terminally, modified isoforms p53β and p53γ (Fig. 1A; [23]), we analyzed recombination on chromosomally integrated EGFP-substrate (Supplementary Fig. S1A). p53α expression in the p53α-negative K562*(HR-EGFP/3*′*EGFP)* reporter cells [19, 24–26] stimulated recombination 3.7-fold compared to control samples (ctrl) (Fig. 1B, C). To the contrary, expression of all other p53-isoforms did not alter recombination frequencies compared to ctrl (Fig. 1B, C). Conditions of protein expression were based on preceding titration experiments to ensure comparable p53 levels as shown in the right panels of Fig. 1B, C. Notably, while p53α-expressing cells stimulated p21 and to a lesser extent also MDM2 expression in K562 cells, alternative p53-isoforms failed to induce p21 (Fig. 1B, C). In conclusion, both N-terminally truncated and C-terminally modified p53-isoforms were impaired in activating homology-directed DDT.

### p53-isoforms differentially affect DNA replication dynamics after mock- and MMC-treatment

**Fig. 2:**
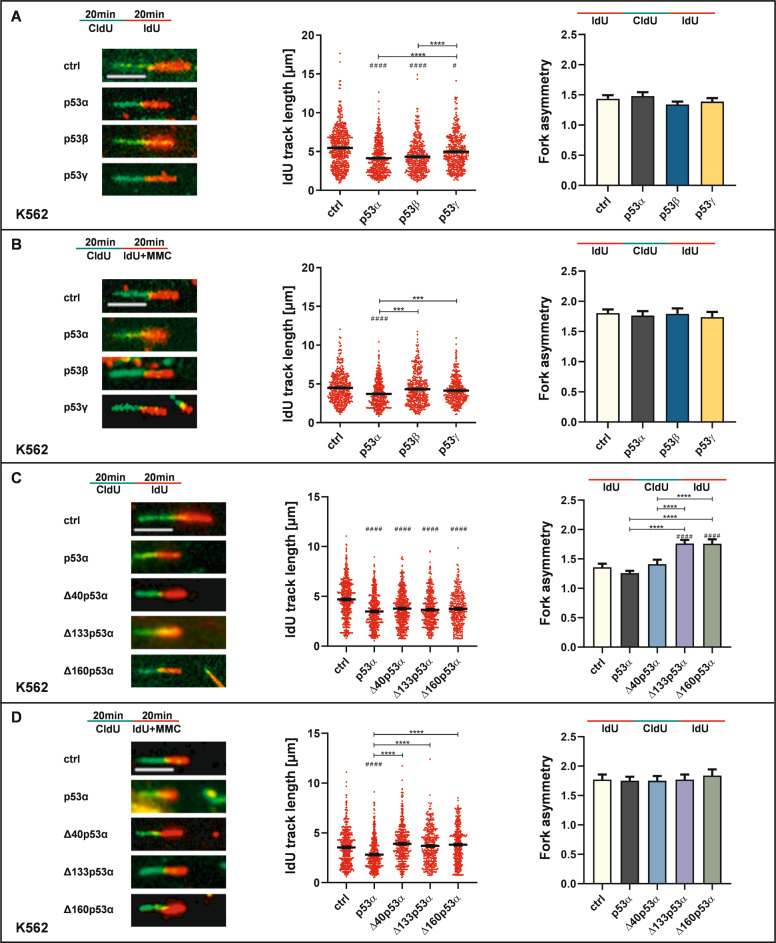
p53α and its isoforms modulate nascent DNA synthesis. K562 cells were transfected with expression plasmids for p53α, alternative p53 isoforms [**A**, **B** p53β, p53γ or **C**, **D** Δ40p53α, Δ133p53α, Δ160p53α] or EV in controls (ctrl). Forty-eight hours after transfection DNA fiber spreading assay was performed. Graphic overviews in the left panel show the experimental outline and representative fibers. Cells were subsequently incubated with 5-chloro-2′-deoxyuridine (CldU, 20 µM) and 5-iodo-2′-deoxyuridine (IdU, 200 µM) for 20 min. During IdU-incorporation cells were either mock-treated (**A**, **C**) or treated with 3 µM MMC (**B**, **D**). Both, CldU- and IdU-tracks were measured but for clarity graphic presentations in the middle panel focus on IdU-tracks in ongoing forks (≥361 to ≥423 fibers in two independent biological experiments). Right panels show the graphic presentation of FA with the respective schematic overview on top. DNA fibers were reanalyzed comparing track lengths of IdU incorporation (red) originating from the same CldU track (green). Graphs represent ≥66 to ≥121 fibers from two independent biological experiments. For graphic presentation, calculation of SEM, and statistically significant differences GraphPadPrism8.4 software was used. Statistically significant differences among groups were calculated by Dunn’s multiple comparisons test. # indicates a statistically significant difference between ctrl and the respective p53 isoform. (#)*P* < 0.05, ***P < 0.001, ****(# # # #), *P* < 0.0001 (scale bar: 5 µm).

Activation of homology-directed DDT by p53α has been linked to a replication slow-down detectable by DNA fiber spreading assay [19, 20]. Accordingly, we investigated the effect of the p53-isoforms on DNA replication speed in K562 cells (Fig. 2). After sequential incubation with 5-Chloro-2-deoxyuridine (CldU) and 5-iodo-2-deoxyuridine (IdU) (experimental overview and representative fibers in left panels of Fig. 2), we monitored a similar shortening of the DNA track lengths after expression of p53α or p53β compared to ctrl after mock-treatment (Fig. 2A). Expression of p53γ generated intermediate track lengths. After treatment with the replication stress-inducing agent MMC only p53α, but not p53β or p53γ, decreased track lengths (Fig. 2B). To investigate, if deceleration of DNA replication was associated with stalling, we measured the ratio of two IdU track lengths emanating from the same CldU track (fork asymmetry [FA]; graphic presentations shown on top of right panels in Fig. 2) [19, 27]. While MMC-treatment increased FA, no further changes were seen after p53α, p53β, or p53γ expression (Fig. 2A, B).

Next, we performed DNA fiber spreading assays after the expression of p53α- or ΔN-isoforms. Similar as with the C-terminal isoforms, we observed differences between mock- and MMC-treatments: after mock-treatment expression of p53α and all ΔN-isoforms decelerated DNA replication (Fig. 2C). Interestingly, FA analysis revealed increased values in Δ133p53α- and Δ160p53α-expressing cells compared to all other samples. After MMC-treatment only p53α shortened DNA track lengths, while FA values did not differ between the samples (Fig. 2D). Hence, whereas track shortening in Δ133p53α and Δ160p53α-expressing cells was associated with stalling after mock-treatment, shortening in p53α or Δ40p53α-expressing cells did not, compatible with a role in continuous deceleration of nascent DNA synthesis. As such a role of p53α was demonstrated to depend on POLι [19], we examined POLι expression levels in K562 cells after mock- and MMC-treatment. However, no significant differences were observed (Supplementary Fig. S1B, C).

To examine nascent DNA synthesis as a function of the p53-isoforms in another p53-negative cell type, we engaged Saos-2 (Supplementary Fig. S2). Here, p21 and MDM2 were detectable in p53α-expressing cells only (Supplementary Fig. S2A). Results from DNA fiber spreading assays were consistent with the results in K562 except that in mock-treated cells p53γ was not only intermediate, but fully functional in track shortening (Supplementary Fig. S2B) and Δ40p53 only partially functional (Supplementary Fig. S2D).

Since the p53 targets p21 and MDM2 were reported to play roles in the regulation of replication [28–30], we expressed exogenous p21 and MDM2 in cells with representative C-terminal and ΔN-isoforms. DNA fiber spreading analyses revealed that these proteins did not reconstitute the replication track shortening phenotype of p53α in p53γ, Δ133p53α or Δ160p53α-expressing cells treated with MMC (Supplementary Fig. S3). From this, it is unlikely that changes in p21 and MDM2 expression explain dysfunction of p53-isoforms in the p53-POLι DDT-pathway.

Taken together, C-terminal and ΔN-isoforms of p53 show at least partial function after mock- but full loss-of-function after MMC-treatment in track shortening. Therefore, failure to decelerate replication under conditions of enforced replication stress mirrors their defect in recombination stimulation (Fig. 1B, C) indicating loss of key biochemical activities in the homology-directed DDT-pathway.

### C-terminal and ΔN-isoforms no longer promote ternary p53-POLι-PCNA complex formation in MMC-treated cells

**Fig. 3:**
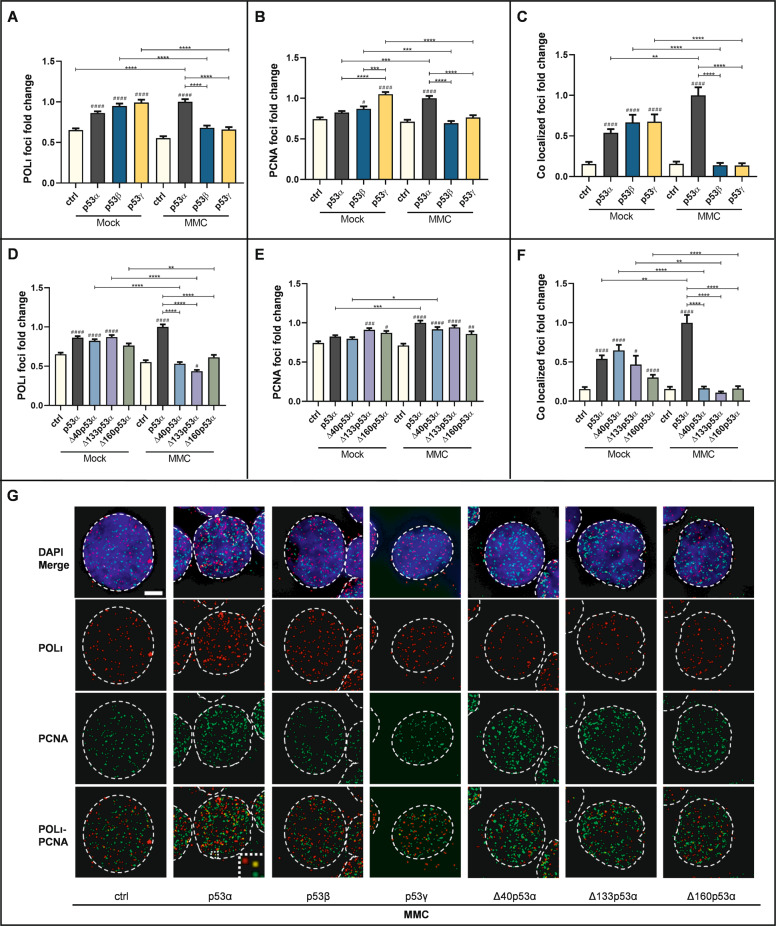
Effect of the p53 isoforms on the subcellular distribution and association of POLι and PCNA. K562 cells were transfected with expression plasmids for p53α, alternative p53 isoforms [**A**−**C** p53β, p53γ, **D**−**F** Δ40p53α, Δ133p53α, Δ160p53α] or EV in controls (ctrl). Forty-eight hours after transfection, cells were mock- or MMC-treated (3 μM, 45 min, 3 h release) and processed for immunostaining to visualize POLι and PCNA foci as well as their co-localization. At least 100 nuclei were scored in ≥ two independent experiments. Mean values for p53α expressing cells after MMC-treatment were set to 1 [on average 103 POLι (**A**, **D**), 133 PCNA (**B**, **E**), and 15 POLι-PCNA co-localized (**C**, **F**) foci/nucleus]. For graphic presentation, calculation of SEM and statistically significant differences via Dunn’s multiple comparisons test GraphPadPrism8.4 software was used. Representative images of MMC-treated samples are shown in (**G**). The experiments shown in (**A**) and (**D**), (**B**) and (**E**) as well as (**C**) and (**F**) were performed together (values of ctrl and p53α are identical but separated in different panels for clarity) and with the experiments presented in Fig. 6. *(#)*P* < 0.05, **(# #)*P* < 0.01, ***(# # #)*P* < 0.001, ****(# # # #), *P* < 0.0001 (scale bar: 5 µm).

Our previous work demonstrated that POLι forms a complex with p53 and PCNA and that this p53-POLι idling complex is required to slow down replication [19, 20]. Therefore, we investigated the subnuclear distribution of POLι and PCNA as well as their association after the expression of different p53-isoforms in K562 (Fig. 3). In mock-treated cells, we observed increased accumulation of POLι-foci in all p53-expressing cells except for Δ160p53α and of PCNA-foci except for p53α and Δ40p53α (Fig. 3A, B, D, E). Moreover, we observed a 2- to 4-fold increase of co-localized POLι-PCNA-foci in all p53-expressing cells (Fig. 3C, F). However, after MMC-treatment, increased numbers of POLι- as well as co-localized POLι-PCNA-foci seen after p53α expression was lost with all other p53-isoforms (Fig. 3A, C, D, F). This defect was similarly seen regarding PCNA-foci in cells expressing C-terminal p53-isoforms but not ΔN-isoforms (Fig. 3B, E). Specific POLι detection was verified by POLι-silencing (Supplementary Fig. S4). Moreover, we analyzed the percentages of MMC-treated K562 cells in different cell cycle phases and in apoptosis as indicated by a sub-G1 content (Supplementary Fig. S5). This analysis did not reveal significant differences in cells expressing different p53-isoforms, though a trend of enhanced apoptosis was noticed in p53α-expressing cells.

**Fig. 4:**
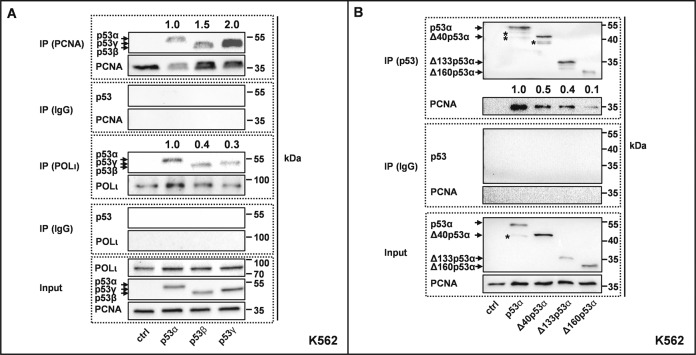
p53 isoforms differently interact with PCNA and POLι. K562 cells were transfected with expression plasmids for p53α, alternative p53 isoforms [**A** p53β, p53γ, **B** Δ40p53α, Δ133p53α, Δ160p53α] or EV in controls (ctrl). Forty-eight hours after transfection, cells were harvested for immunoprecipitations (IP) after preparation of crosslinked chromatin (**A**) or without crosslinked chromatin preparation (**B**). For the pull-downs of PCNA (**A**) anti-PCNA antibody (mouse, ab29, abcam, Cambridge, UK) or control Mouse IgG (Santa Cruz, Dallas, Texas, USA), for the pull-downs of POLι (**A**) POLι-antibody (rabbit, A301-304A, Bethyl, Montgomery, USA) or Rabbit IgG were used. For the pull-down of p53 (**B**) a mix of anti-p53(DO11) (mouse, MCA1704, BioRad Laboratories, München, Germany) and anti-p53(Pab421) (mouse, OP03, Calbiochem, Darmstadt, Germany) or control Mouse IgG were used. Subsequent immunoblotting relied on anti-POLι (rabbit, A301-304A, Bethyl, Montgomery, USA), anti-p53(DO1) (mouse, mAb, 554293, BD, Biosciences, Franklin Lakes, New Jersey, USA) in (**A**), anti-p53(DO11) (mouse, GTX75258, Genetex, Irvine California, USA), anti-p53 (PAb421, rabbit, ab245685, Abcam Cambridge, UK), anti-PCNA (mouse, ab29, abcam, Cambridge, UK) in (**B**) and light chain-specific peroxidase-coupled secondary antibody. *, asterisks mark bands possibly stemming from proteolytic cleavage of the p53-isoform. Quantification of band intensities of co-precipitated proteins relative to their input is indicated. Representative Western blots from three to four experiments are shown.

Next, we investigated physical interactions between p53, POLι, and PCNA in co-immunoprecipitation studies after the preparation of crosslinked chromatin. After pull-down of POLι, we detected less than half of the input levels of p53β and p53γ in the co-precipitate as compared with p53α (Fig. 4A). In PCNA co-precipitates p53β and p53γ levels were not reduced. After pull-down of p53α without crosslink, we also observed an interaction with PCNA, as reported before [19]. However, PCNA band intensities were reduced by 50−90% relative to input in co-precipitates of the N-terminally truncated p53-isoforms (Fig. 4B). Previously, we showed that p53α binds POLι via its N-terminus [20]. Consistently, p53α but not Δ40p53α co-immunoprecipitated POLι (Supplementary Fig. S6). Altogether, all alternative p53-isoforms are defective in supporting the formation of POLι-PCNA-complexes after MMC-treatment in situ. In co-precipitation experiments, POLι-p53-complex formation is defective in cells expressing any alternative isoform and PCNA−p53 interactions are compromised in cells expressing ΔN-isoforms of p53.

### Regulation of PCNA ubiquitination by p53-isoforms

**Fig. 5:**
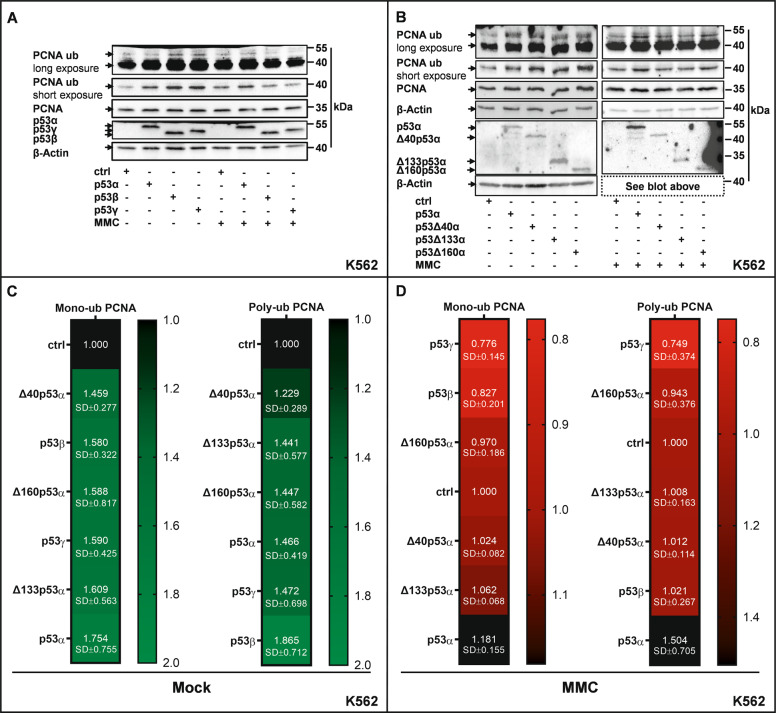
Altered PCNA ubiquitination after expression of p53α or alternative isoforms. K562 cells were transfected with expression plasmids for p53α, alternative p53 isoforms [**A** p53β, p53γ, **B** Δ40p53α, Δ133p53α and Δ160p53α] or EV in controls (ctrl). Forty-eight hours after transfection, cells were either mock- or MMC-treated (3 μM, 45 min, 3 h release), subsequently cells were lysed with either lysis buffer for protein extraction (50 mM Tris [pH7.4], 150 mM NaCl, 2 mM EGTA, 2 mM EDTA, 25 mM Sodium fluoride, 25 mM β-Glycerol phosphate, 0.1 mM Sodium vanadate, 0.2% Triton X-100, 0.3 % Nonidet P40, complete protease inhibitor [Roche]) or IP lysis buffer (50 mM Tris-HCl [pH 8], 150 mM NaCl, 1% NP40, complete protease inhibitor [Roche]) and then processed for immunoblotting using ubiquityl PCNA (Lys164, D5C7P, Cell Signaling, Massachusetts, USA) antibody, recognizing PCNA protein only when ubiquitinated at Lys164, as well as antibodies against PCNA and p53. β-Actin was used as loading control. “ub” indicates ubiquitination. Quantification of respective protein expression level was carried out using ImageLab software. Levels of PCNA mono-/polyubiquitination were corrected for PCNA and normalized to ctrl (**C**, **D**) which was set to 1 on each blot. Statistically significant differences among groups were calculated by Friedman test followed by Wilcoxon-signed ranks test in case of statistical significance. Representative Western Blots from cells expressing ctrl, p53α, and C-terminal isoforms (**A**) or cells expressing ctrl, p53α, and ΔN-isoforms (**B**). Heatmap of mean values from ≥ 5 independent experiments (**C**) or ≥ 4 independent experiments (**D**).

Deceleration of nascent DNA synthesis in the p53-POLι DDT-pathway buys time for the cell to polyubiquitinate PCNA and recruitment of helicase-like transcription factor (HLTF) and Zinc Finger Ran-Binding Domain-Containing Protein 3 (ZRANB3) to bypass the replication barrier [19, 20]. Western Blot analysis revealed a statistically significant 1.5- to 1.8-fold increase (*P* ≤ 0.0156) of PCNA-monoubiquitination in mock-treated cells after expression of any of the p53-isoforms (Fig. 5A–C). PCNA-polyubiquitination showed a similar pattern (Fig. 5A–C), but the statistical significance of the increase in polyubiquitination was reached only with p53α-expressing cells (*P* = 0.0156). After MMC-treatment we detected reduced levels of mono- and polyubiquitinated PCNA in cells expressing C-terminal or ΔN-isoforms as compared with p53α (Fig. 5A, B, D), which reached statistical significance (*P* = 0.0078) with p53β and p53γ samples (Fig. 5D). We conclude that alternative p53-isoforms are compromised in supporting PCNA-ubiquitination after MMC-treatment.

Subsequently, we silenced HLTF and ZRANB3 to investigate a potential impact on PCNA-ubiquitination (Supplementary Fig. S7) as both proteins were found to exert critical roles in the POLι DDT-pathway, most likely mediating PCNA-polyubiquitination and fork reversal, respectively [19]. In MMC-treated cells expressing p53α, we found that knockdown of HLTF reduced PCNA-poly- but not -monoubiquitination (Supplementary Fig. S7A, C, E), while knockdown of ZRANB3 had no effect (Supplementary. Fig. S7B, D, F). In MMC-treated cells, neither HLTF nor ZRANB3 affected PCNA-polyubiquitination after p53γ or Δ133p53α expression strengthening the notion of their reduced capacity to stimulate PCNA-polyubiquitination.

### Alternative p53-isoforms exert an inhibitory effect on p53α in the p53-POLι DDT-pathway

**Fig. 6:**
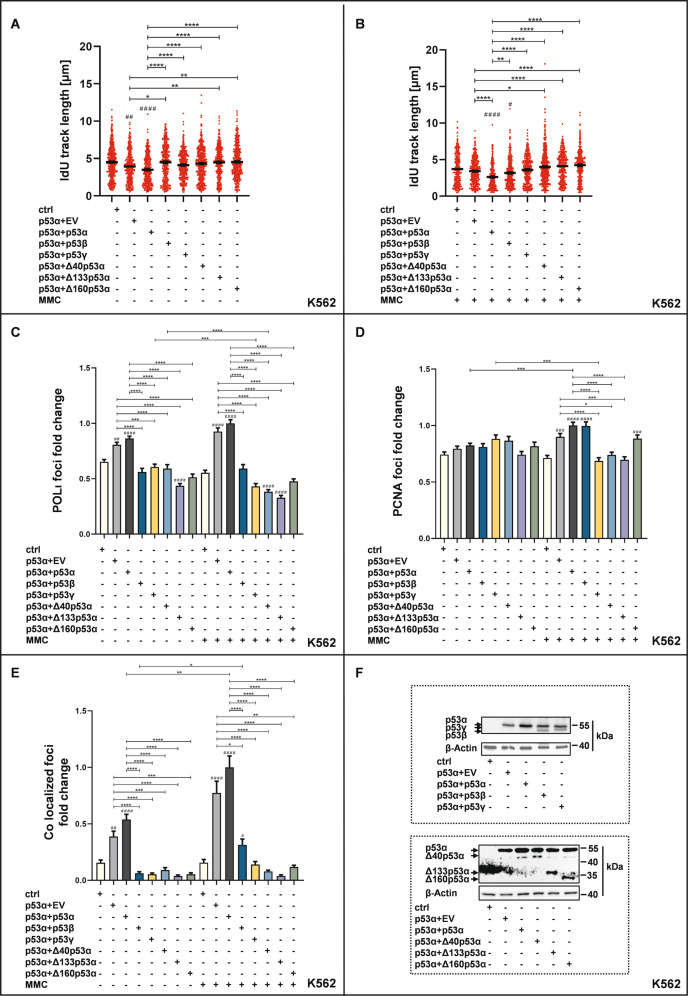
Co-expression of p53 isoforms affects the POLι-dependent DDT pathway. K562 cells were transfected with a total amount of 10 μg plasmid DNA, containing either EV only in controls (ctrl) or expression plasmid for p53α plus EV (p53α + EV) or for p53α plus p53α (p53α + p53α) or for p53α plus one of the alternative isoforms (p53α + p53β, p53α + p53γ, p53α + Δ40p53α, p53α + Δ133p53α, and p53α + Δ160p53α). For graphic presentation, calculation of SEM and statistically significant differences via Dunn’s multiple comparison test GraphPadPrism8.4 software was used. # indicates a statistically significant difference between ctrl and the respective p53-isoform values. *(#)*P* < 0.05, **(# #)*P* < 0.01, ***(# # #)*P* < 0.001, ****P(# # # #) < 0.0001. **A, B**: DNA fiber-spreading assays were performed 48 h after transfection. The experimental design was as described in the legend to Fig. 2. Both mock-treated CldU- and IdU-tracks were measured but for clarity graphic presentations focus on IdU-tracks in ongoing forks (≥421 fibers (**A**) and ≥320 fibers (**B**) in two independent biological experiments). POLι (**C**), PCNA (**D**), POLι-PCNA colocalization (**E**) foci fold changes in K562 cells revealed by immunofluorescence microscopy. Mean values of p53α + p53α expressing and MMC-treated samples were set to 1 [on average 103 POLι (**C**), 133 PCNA (**D**), 15 POLι-PCNA colocalization (**E**) foci per nucleus]. Experiments were performed together with the ones shown in Fig. 3. (**F**) Western blots for co-expression of p53 isoforms. Co-expression of p53α and C-terminal isoforms were detected by anti-p53 antibody DO-1 (mouse, 554293, BD Biosciences, Franklin Lakes, New Jersey, USA). Co-expression of p53α and ΔN-terminal isoforms were detected by anti-p53 antibody DO-11 (mouse, GTX75258, Genetex, Irvine California, USA).

Previous reports described the inactivation of p53α by hetero-oligomerization with alternative p53-isoforms [31–33]. Consequently, we were interested in how co-expression of p53α with the different p53-isoforms will affect the p53-POLι-dependent DDT. In mock-treated cells replication slow-down was observed after expression of p53α at a level of 50% (p53α plus empty vector, p53α + EV) and 100% (p53α + p53α) versus ctrl, but lost after co-expression (50%:50%) of p53α with one of the alternative isoforms (Fig. 6A, F). After MMC-treatment we observed replication deceleration in p53α + p53α but not in p53α + p53γ, p53α + Δ40p53α, p53α + Δ133p53α or p53α + Δ160p53α samples versus ctrl and an intermediate phenotype in p53α + p53β samples (Fig. 6B). Importantly, comparing track lengths in samples co-transfected with p53α + alternative isoforms versus p53α + p53α provided evidence for loss-of-function, i.e., loss of the track shortening function in all isoforms independently of treatment (Fig. 6A, B).

Notably, a 50% expression level of p53α in p53α + EV samples was sufficient to decelerate replication versus ctrl in unperturbed but not in MMC-treated cells (compare p53α + EV with p53α + p53α in Fig. 6A, B). Most interestingly, when comparing p53α + EV samples with samples co-transfected with p53α + alternative isoforms, we found longer tracks in p53α + p53β, p53α + Δ133p53α, and p53α + Δ160p53α samples after mock-treatment and in p53α + Δ40p53α, p53α + Δ133p53α, and p53α + Δ160p53α samples after MMC-treatment. These observations suggested an inhibitory effect, which was most pronounced in p53α + Δ133p53α and p53α + Δ160p53α samples.

Strikingly, we obtained almost the same results in Saos-2-cells: Co-expression of each alternative p53-isoform did not decelerate nascent DNA synthesis as seen in p53α + p53α samples versus ctrl independently of treatment, supporting the loss-of-function concept (Supplementary Fig. S8A, B). Moreover, compared with p53α + EV longer tracks were measured in p53α + Δ133p53α mock-treated cells and p53α + p53β, p53α + Δ133p53α, and p53α + Δ160p53α MMC-treated cells. These Saos-2 data strengthened the concept of an inhibitory effect of the alternative p53-isoform Δ133p53α in particular.

Next, we also investigated the subnuclear distribution of POLι and PCNA, as well as their co-localization after co-expression of p53α and alternative isoforms (Fig. 6C−E). Both, after mock- and MMC-treatment, co-expression of p53α together with any alternative p53-isoform reduced accumulation of POLι and POLι-PCNA colocalizing foci observed after expression of p53α alone (Fig. 6C, E). It also abrogated p53α-mediated PCNA-foci accumulation after MMC-treatment in p53α + p53γ, p53α + Δ40p53α, and p53α + Δ133p53α samples (Fig. 6D). Notably, p53α + p53β co-expression causing intermediate DNA track lengths (Fig. 6B**)** also showed intermediate enhancement of POLι-PCNA-co-localization after MMC-treatment (Fig. 6E). Altogether, co-expression of alternative isoforms blocked p53α-mediated recruitment of POLι into PCNA-complexes and consequently slow-down of DNA replication, which was still observed in mock-treated cells expressing individual isoforms. In conclusion, co-expression of p53-isoforms compromises the p53-POLι DDT not only via loss-of-function but also via inhibitory effects.

**Fig. 7:**
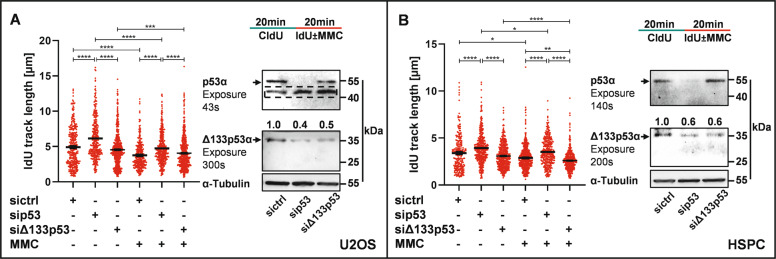
Knockdown of endogenous p53-isoforms induces changes in fiber track lengths. U2OS cells (**A**) or HSPCs (**B**) were transfected with nonsense siRNA (sictrl), siRNA targeting all p53-isoforms (sip53) or siRNA targeting Δ133p53/Δ160p53 isoforms (siΔ133p53). **A, B**: 24 h after transfection, cells were sequentially incubated with CldU (20 µM) and IdU (200 µM) for 20 min. During IdU-incorporation, cells were either mock-treated or treated with 3 µM MMC. Both CldU- and IdU-tracks were measured while only IdU-tracks in ongoing forks (≥257 fibers from two independent biological experiments) are graphically presented for clarity. Knockdown of p53-isoforms was verified by immunoblot staining using anti-p53 (DO-11, MCA1704, Biorad) shown in the right panel of (**A, B**) each. Quantification of Δ133p53α relative to α-Tubulin levels was achieved by Imagelab and is indicated above the blots. The dashed frame in (**A**) marks an unspecific band stained by DO-11 in U2OS cells that had to be cut out, followed by antibody reincubation and a long exposure for 300 s to permit immunodetection of the Δ133p53α-band. Statistically significant differences between groups were calculated by Dunn’s multiple comparisons test. *P < 0.05, **P < 0.01, ***P < 0.001, ****P < 0.0001.

To test the influence of endogenously expressed p53-isoforms, we performed silencing experiments engaging siRNA targeting either all p53 isoforms or siRNA targeting Δ133p53α (and Δ160p53α), i.e., the isoform most robustly affecting DNA replication speed in the presence of p53α (Fig. 6A, B and Supplementary Fig. S8A, B). We chose U2OS cells, as well as primary hematopoietic stem and progenitor cells (HSPCs) derived from human cord blood, as both carry wild-type *TP53* and express p53α and Δ133p53α (Fig. 7A, B). When performing DNA fiber spreading assays, we found that DNA replication was accelerated both in mock- and MMC-treated U2OS cells and HSPCs after silencing all p53-isoforms (Fig. 7A, B). Selective knockdown of Δ133p53/Δ160p53 (siΔ133p53) caused replication slowing in MMC-treated HSPCs (Fig. 7B), matching lack of replication deceleration after ectopic expression of Δ133p53α or Δ160p53α as compared to p53α in MMC-treated K562 cells (Fig. 2D). For comparison, replication track lengths were unaffected by siΔ133p53 in U2OS cells, most likely due to a lower Δ133p53α/p53α ratio as compared to HSPCs as deduced from the exposure times necessary to detect p53α and Δ133p53α in immunoblots (Fig. 7A, B). Altogether, the data in HSPCs confirmed an antagonistic role of endogenous Δ133p53 towards the DNA replication decelerating role of p53α.

## Discussion

In this study, we provide evidence that both C-terminal and ΔN-isoforms show loss of the p53α-specific functions in the p53-POLι-dependent DDT-pathway [19, 20]. Underscoring the biological relevance, we find interference of alternative p53-isoforms, particularly of Δ133p53α and Δ160p53α, with p53α′s functions in DNA replication both in human tumor and stem cells.

### Alternative p53-isoforms show loss-of-function in the p53-POLι–dependent DDT-pathway mediating homology-directed bypass of replication barriers

Treatment with MMC causes DNA intra- and interstrand-crosslinks, which represent the ultimate roadblock for nascent DNA synthesis [34, 35]. The p53-POLι-dependent DDT-pathway can overcome these obstacles [20]. Hallmarks of this pathway, namely (i) deceleration of DNA replication, (ii) increased POLι-foci assembly and PCNA-POLι-co-localization, as well as (iii) PCNA-ubiquitination were absent in cells expressing alternative p53-isoforms after MMC-treatment. Yet, under unperturbed growth conditions, these key functions were retained at least partially. Another hallmark, namely replication-associated recombination was undetectable even in untreated cells expressing alternative p53-isoforms. Such sensitive detection of loss-of-function can be explained because this approach selectively identifies homology-directed bypass at hard-to-replicate sites including extended secondary structures or DNA-crosslinks stemming from naturally occurring aldehydes [21, 36]. For comparison, DNA Fiber Spreading Assays reflect nascent DNA synthesis at each progressing replication fork all over the genome irrespective of a barrier, why enforced replication stress is required to uncover defects.

**Fig. 8:**
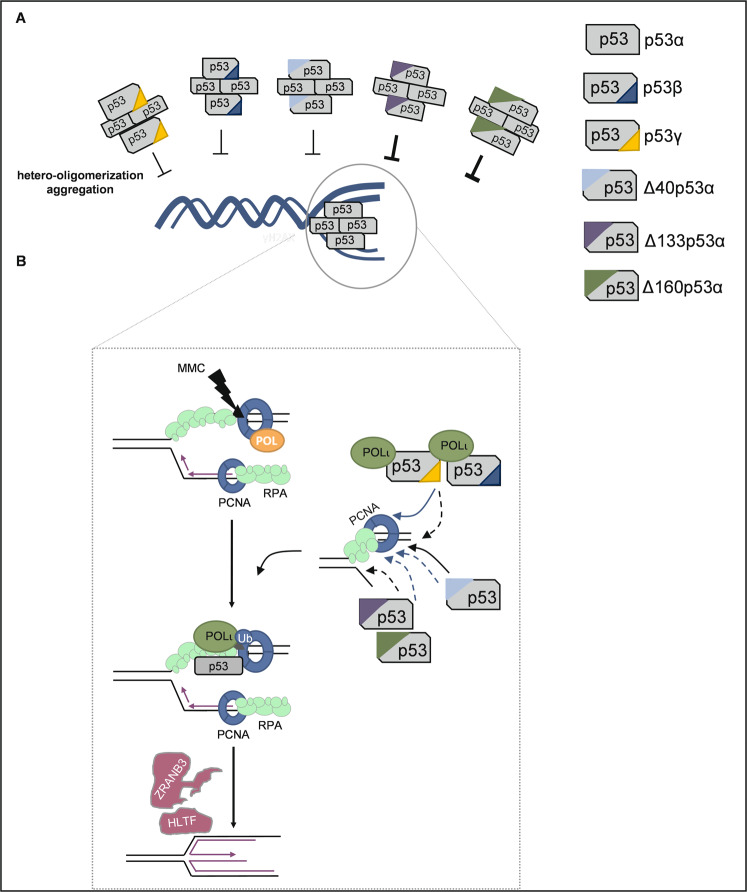
Model for the impact of different p53 isoforms on POLι-dependent DDT. **A** Hetero-oligomerization or aggregation of p53α with alternative p53 isoforms, particularly with p53α + Δ133p53α or p53α + Δ160p53α, can antagonize p53α functions in the POLι-dependent DDT. **B** C-terminal p53 isoforms still interact with PCNA [20, 41] but are compromised in binding to three-stranded DNA junctions like replication forks due to shortened OD [38]. Intact DBD, OD, and RPA-interaction sites [8], but compromised interactions with POLι and PCNA due to lack of the N-terminal end in Δ40p53α permit limited replication fork recognition only. Δ133p53α or Δ160p53α are compromised in DNA-binding due to the N-terminally truncated DBD and in RPA-, POLι and PCNA-binding due to the missing N-terminus. However, all N-terminal p53 isoforms retain the OD, enabling hetero-oligomerization with p53α and therefore dominant-negative interference with p53α functions in performing idling events in complex with POLι and PCNA, giving time for PCNA ubiquitination and FR events by HLTF and ZRANB3 [19]. Black arrows, DNA-binding; blue arrows, PCNA-binding; stippled arrow, compromised interaction.

Since alternative p53-isoforms still promoted DNA replication slow-down and PCNA-POLι-co-localization under unperturbed conditions, we conclude that alternative p53-isoforms can build p53-PCNA-POLι-complexes at replication sites at least conditionally. Interestingly, these intermediate DDT-pathway defects originate from different combinations of deficiencies and proficiencies of key biochemical activities linked with specific protein domains (Fig. 8). In case of C-terminal p53-isoforms, both p53β and p53γ possess an intact N-terminus and consequently still interacted with PCNA and partially with POLι in immunoprecipitation experiments [20]. p53β and p53γ also possess an intact DBD [23] and retain the interaction site with topoisomerase-I (aa 302-321 [37]), another critical interaction partner in the pathway [20, 25]. However, they lost half of the OD, which is required to enhance binding to three-way DNA junctions in the tetramer conformation [38] and accordingly to trigger p53-POLι-dependent DDT [20]. p53β was reported to still bind DNA, however, in a dimeric conformation [39]. We propose that C-terminal p53-isoforms are transiently recruited to replication sites via physical interactions with DDT factors. This may suffice to promote PCNA-ubiquitination (a pre-requisite for DDT [40]) and deceleration of nascent DNA synthesis under unperturbed conditions, but no longer when MMC-roadblocks require tight association to DNA.

The ΔN-isoforms possess an intact OD and more than two-thirds of the DBD. They are completely devoid of the PCNA and POLι interaction sites within the N-terminal 40 aa of p53α [20, 41]. Consistently, we observed that Δ40p53α was defective in co-immunoprecipitating these two proteins. ΔN-isoforms can no longer contact the basal transcription machinery through the TAD I [42]. As a transcriptional activator, p53 prominently upregulates p21 and MDM2 [43–45], i.e., two proteins, which exhibit p53-independent functions in DNA replication [29, 30, 46]. Here, we confirmed reduced p21 and MDM2 levels in cells expressing alternative p53-isoforms, particularly in p53γ, Δ133p53α, and Δ160p53α-expressing cells. However, re-expression of p21 and MDM2 did not rescue deceleration of nascent DNA synthesis, excluding that the p53 target gene products are the missing components in cells expressing alternative p53-isoforms.

The p53-isoforms Δ133p53α and Δ160p53α were special in that they showed signs of a replication defect already under unperturbed conditions. Thus, we noticed increased replication fork stalling (RFS) in the presence of these p53-isoforms to the extent elicited by MMC-treatment. Shortening of DNA tracks can be explained by exonucleolytic degradation, continuous slow-down of DNA synthesis or by RFS [19]. We, therefore, propose that replication deceleration observed in cells expressing Δ133p53α and Δ160p53α is due to increased RFS and not due to p53α-POLι-dependent DDT [19]. As this pathway is inactive in cells expressing exonuclease-deficient p53(H115N) [47], it was proposed that p53-POLι complexes slow down DNA synthesis via iterative idling that gives time for HLTF-mediated PCNA-polyubiquitination and ZRANB3-mediated fork reversal, i.e., homology-directed bypass of the barrier [19]. Compatible with loss of idling, Δ133p53α and Δ160p53α not only lack the N-terminal PCNA- and POLι-interaction site, but additionally and distinct from Δ40p53α, the binding site (aa 38-58) for human single-stranded DNA-binding protein RPA [9, 48] and parts of the DBD enabling specific recognition and exonucleolytic attack of three-way junctions [11, 38]. Given the propensity of Δ133p53α and Δ160p53α to form aggregates [39], it is conceivable that these p53-isoforms non-productively occupy DNA replication sites through the residual DBD and OD supported by the C-terminus recognizing DNA lesions [10, 49] and by interacting with topoisomerase I [37, 50] and PARylated PARP1 [51]. In support of the view that Δ133p53α and Δ160p53α interact with replication forks, we noticed an increase of PCNA foci without a concomitant rise in POLι-PCNA colocalizing foci. Additionally, Δ133p53α and Δ160p53α can no longer compete for RPA70 subunit-binding with other potentially harmful proteins such as the nuclease MRE11, known to act on stalled and deprotected forks [19, 52–55].

Altogether, p53β, p53γ and Δ40p53α display residual performance in the p53-POLι-dependent DDT-pathway, as at least one of the underlying biochemical activities are retained each (Fig. 8). However, Δ133p53α and Δ160p53α, with full proficiency to form tetramers, but devoid of regions providing specificity for this DDT-pathway cause non-specific deceleration of DNA replication by RFS.

### Alternative p53-isoforms exert an inhibitory effect in the p53α-POLι-dependent DDT-pathway, which impacts on tumor suppressor functions

Mutant p53 proteins were described to experience loss-of-function, acquire gain-of-function and interfere dominant-negatively with canonical and non-canonical wild-type p53 functions through hetero-oligomerization and aggregation [56–60]. Squelching out of factors required for wild-type p53 function represents even one further dominant-negative mechanism. In this work we found three pieces of evidence indicating inhibitory effects of alternative p53-isoforms. First, all alternative isoforms abrogated DNA replication slow-down by p53α under unperturbed conditions. Second, Δ133p53α and Δ160p53α elongated DNA tracks after ectopic co-expression with p53α in tumor cells. Third, silencing endogenous Δ133p53/Δ160p53 shortened tracks in MMC-treated HSPCs.

A dominant-negative effect of ΔN-isoforms with intact OD could be explained by the formation of mixed hetero-oligomers with less specific DNA-binding properties [32], as the N-terminal TAD of p53 is known to directly interact with the DBD promoting specific DNA interactions [61]. Having shown that Δ133p53α and Δ160p53α cause RFS already on their own, we propose that these ΔN-isoforms additionally squelch out C-terminal p53α-interaction partners.

Accumulating evidence has shown that an increased ratio of ΔN-isoforms/p53α is associated with poorer overall survival or cancer aggressiveness, particularly for Δ133p53 in esophageal squamous cell carcinoma [62], Cholangiocarcinoma [63], and prostate cancer [64]. Here, we showed, that alternative p53-isoforms, Δ133p53α and Δ160p53α, in particular, interfere with co-expressed p53α in DDT. Since homology-directed DDT-pathways, involving HLTF- and ZRANB3-mediated fork reversal, protect the genome from unrestrained and mutagenic bypass mechanisms like TLS [65, 66], we anticipate that these isoforms increase genomic instability. Besides, the same ΔN-isoforms stimulate proliferation and inhibit apoptosis [15, 23], all-in-all creating a dangerous combination of pro-mutagenic and pro-survival features, known to drive carcinogenesis. Intriguingly, p53α evolved from N-terminally truncated Δ40p53α [15]. Hence, p53 gained the N-terminus which is critical for its canonical transcriptional activities but also for its non-canonical function in regulating replication dynamics by interacting with PCNA and POLι [20]. Therefore, our observations may serve to better understand the role of p53-isoforms in cancer and stem cells regarding the maintenance of human genome stability.

## Materials and methods

### Cell culture and drug treatment

K562 and K562(*HR-EGFP/3*′*EGFP*) cells were cultivated in RPMI1640 medium (Gibco by Thermo Fisher Scientific, Waltham, Massachusetts, USA) supplemented with 13% FBS (Merck Millipore Darmstadt, Germany) and 1.3% Penicillin-Streptomycin-Glutamine (Gibco by Thermo Fisher Scientific, Waltham, Massachusetts, USA). Saos-2 cells were cultivated in McCoy’s 5A (Modified) Medium (Gibco by Thermo Fisher Scientific, Waltham, Massachusetts, USA) or DMEM medium supplemented with 10% FBS (Merck Millipore Darmstadt, Germany) and 1% Penicillin-Streptomycin-Glutamine (Gibco by Thermo Fisher Scientific, Waltham, Massachusetts, USA). U2OS cells were cultured in DMEM Medium (Gibco by Thermo Fisher Scientific, Waltham, Massachusetts, USA) supplemented with 10% FBS and 1% Penicillin-Streptomycin-Glutamine. For DNA crosslinker treatment cells were washed, incubated with DNA crosslinker Mitomycin C (MMC) (Sigma-Aldrich by Merck, Darmstadt, Germany) containing media at a final concentration of 3 µM or mock-treated with the solvent H_2_O for 45 min, washed and re-incubated with fresh media for additional 3 h. Cell cultures were free from mycoplasma contamination as verified by PCR. HSPCs were isolated from cord blood samples and cultivated as described in [22, 67].

### Recombination measurements

To measure recombination frequencies K562 cells with chromosomally integrated recombination substrate, K562(*HR-EGFP/3*′*EGFP*) [24] were subjected to electroporation with expression plasmids for p53α, p53β, p53γ, Δ40p53α, Δ133p53α, and Δ160 or empty vector (ctrl) as detailed in the Figure legends. Recombination frequencies were measured 72 h after transfection by quantification of one million cells from EGFP-positive cells within the life cell-population (SSC/FSC gate). Mean values of recombination frequencies of p53α expressing cells were set to one and absolute mean frequencies are detailed in the Figure legends.

### DNA fiber spreading assay

To measure DNA replication track lengths for assessment of replication speed the DNA fiber spreading technique was performed as detailed in [20]. CldU (5-Chloro-2-deoxyuridine, Sigma-Aldrich by Merck, Darmstadt, Germany) was incorporated for 20 min. After washing, IdU (5-Iodo-2-deoxyuridine, Sigma-Aldrich by Merck, Darmstadt, Germany) was incorporated in ten times higher concentration for another 20 min. During the IdU pulse, cells were either treated with MMC (3 µM, Sigma-Aldrich by Merck, Darmstadt, Germany) or its solvent H_2_O. Then, cells were washed, harvested, and resuspended in PBS. 2500 cells were put on a slide, lysed with 6 μl of 0.5% SDS, 200 mM Tris–HCl, pH7.4, 50 mM EDTA, and incubated at room temperature for 6 min. Afterwards, they were tilted about 20° to allow DNA-spreading via gravity. Subsequently, slides were covered with aluminum foil, air-dried for 6 min, fixed for 5 min with newly prepared 3:1 methanol:acetic acid, air-dried for 7 min, and stored in 70% ethanol at 4 °C. Twenty-four hours later, the slides were processed for denaturation/deproteination in 2.5 N HCl for 1 h, followed by immunofluorescence staining and microscopy. To determine the speed of ongoing replication, the track lengths of bi-colored forks (CldU: green and IdU: red) were measured in the microscopic images using ImageJ. These track lengths in μm obtained during a 20 min pulse each reflect DNA replication fork speed, which can be calculated with the formula: DNA replication fork speed (kb/min) = 2.59 (kb/μm) × track length (μm) ÷ pulse time (min) [20]. For clarity lengths of the red IdU tracks are shown only. For fork asymmetry analysis, track lengths of IdU-incorporation (red) departing from the same origin (CldU, green) were measured and the respective fork asymmetries show the ratio of longer tracks versus shorter track lengths.

### Immunofluorescence staining

K562 cells were spun onto cytospin glass slides. Then pre-extraction (1 min) was performed (300 mM Sucrose, 50 mM NaCl, 20 mM HEPES (pH 7.4), 3 mM MgCl2, 1 mM EDTA, 0.5% (v/v) Triton X-100, H_2_O). Cells were fixed with 3.7% formaldehyde in PBS 3 h after MMC-release. Permeabilization was performed with 0.5% Triton X-100 (Sigma-Aldrich by Merck, Darmstadt, Germany) for 12 min at RT. Blocking unspecific binding sites was performed by use of 5% goat serum in PBS for 1 h. Immunostaining for 1 h at 37 °C was performed with the primary antibodies anti-PCNA PC10 (mouse mAb PC10, #ab29 Abcam Cambridge, UK), anti-Polymerase ι (rabbit, polyclonal, #PA5-29442, Thermo Fisher Scientific, Waltham, Massachusetts, USA) and was followed by an incubation time of 45 min at 37 °C with the secondary antibodies AlexaFluor555 (anti-rabbit, Invitrogen by Thermo Fisher Scientific, Waltham, Massachusetts, USA) or AlexaFluor488 (anti-mouse, Invitrogen by Thermo Fisher Scientific, Waltham, Massachusetts, USA). Immunofluorescence microscopy of nuclear signals was performed with Keyence BZ-9000 microscope (Keyence, Cologne, Germany). Automated quantification of foci was carried out using BZ-II Analyzer software. Intensity threshold and minimal focus size were maintained throughout one set of simultaneously treated and processed samples, both when detecting single green or red foci.

Immunofluorescence staining after DNA fiber spreading assays was performed after blocking with 5% bovine serum albumin (BSA) for 45 min. Following primary antibodies were used: anti-BrdU for detection of IdU (mouse, mAb, clone B44, #347580 BD BioScience, Franklin Lakes, New Jersey, USA) and anti-BrdU for detection of CldU (rat, mAb, clone BU1/75 [ICR1] Novus #NB500-169 Nordenstadt, Germany or Abcam #ab6326 Cambridge, UK) and incubated at room temperature (RT) for 1 h. As secondary antibodies, AlexaFluor555 (anti-mouse, Invitrogen by Thermo Fisher Scientific, Waltham, Massachusetts, USA) or AlexaFluor488 (anti-rat, Invitrogen by Thermo Fisher Scientific, Waltham, Massachusetts, USA) were used (incubation time: 45 min, RT). DNA fibers were imaged with Keyence BZ-9000 microscope (Keyence, Neu-Isenburg, Germany). DNA fiber track lengths were measured with BZ-II Analyzer software or using FiJi (Fiji is just ImageJ) software [ImageJ Wiki, Laboratory for Optical and Computational Instrumentation, University of Wisconsin-Madison, Wisconsin, USA [68].

### (Chromatin-Crosslink) co-immunoprecipitation and expression analysis

To isolate nuclear extracts, cells were incubated for 12 min with cytoskeletal (CSK, 250 mM Sucrose, 25 mM KCl, 1 mM HEPES (pH 7.5), 1 mM EGTA, 1 mM MgCl_2_, H_2_O) buffer with freshly added protease-inhibitors (1 mM phenylmethylsulfonylflouride (PMSF), 1 mM Na3VO4, 0.1 mM dithiothreitol (DTT), protease inhibitor cocktail [Roche, Basel, Switzerland]). By adding 1% MeOH-free formaldehyde (Thermo Fisher Scientific, Waltham, Massachusetts, USA) in PBS for 10 min at RT crosslinking was performed. To stop this reaction, 10 mM ice-cold glycine was added. Cells were lysed for 15 min on ice in RIPA buffer (10 mM Tris-HCl [pH 7.5], 25 mM sodium-fluoride, 20 mM sodium-chloride, 1% sodium-deoxycholate, 1% Nonidet P40, 0.1% SDS, H_2_O; freshly added inhibitors: 1 mM PMSF, 1 mM Na3VO4, 0.1 mM DTT, protease inhibitor cocktail [Roche, Basel, Switzerland]). For disruption, sonification was carried out in the sonification water bath Sonorex (Bandelin electronic GmbH & Co. KG, Berlin, Germany) at the following conditions: high power, 30 s on/30 s off for 7.5 min, and three repeats of this procedure. After centrifugation, the supernatant was used for immunoprecipitation either of p53 with antibodies Pab421 (mouse, OP03, Calbiochem, Darmstadt, Germany) and p53(Do11) (mouse, MCA1704, BioRad Laboratories, München, Germany) or of PCNA with anti-PCNA (mouse, ab29, Abcam, Cambridge, UK) or of POLι with anti-POL ι (rabbit, A301-304A, Bethyl, Montgomery, USA). Isotype control was performed by using normal IgG (mouse, sc-2025, Santa Cruz Biotechnology, Dallas, Texas, USA).

For normal co-immunoprecipitations, cells were lysed in 50 mM Tris, pH 8; 150 mM NaCl; 1% NP40; complete protease inhibitor (Roche, Basel, Switzerland). Protein-extract and protein G Sepharose (PGS) were rotated over night at 4 °C to remove components unspecifically binding to PGS. In parallel, antibody-PGS mixtures were rotated at 4 °C. Afterwards, protein-extracts were separated from PGS by centrifugation and the supernatants were transferred to the antibody-PGS mixtures, followed by rotation at 4 °C for an additional 3 h. After spin-down, precipitated proteins were washed 5 times with lysis buffer and dissolved in SDS-PAGE sample buffer. For Western blot analysis, protein extracts were separated electrophoretically, transferred to membranes, and proteins were immunodetected via chemiluminescence. To detect the protein of interest the following antibodies were used: anti-MDM2 (mouse, MABE281, Merck Millipore Darmstadt, Germany), anti-p21 (mouse, 556430, BD Biosciences, Franklin Lakes, New Jersey, USA), anti-p53 (DO-1, mouse, 554293, BD Biosciences Franklin Lakes, New Jersey, USA), anti-p53 (DO-11, mouse, GTX75258, Genetex, Irvine California, USA), anti-p53 (PAb421, rabbit, ab245685, Abcam Cambridge, UK), anti-POLι (rabbit, A301-303A, Bethyl Montgomery, Texas, USA), anti-PCNA (mouse, ab29, Abcam Cambridge, UK), anti-Ubiquityl-PCNA (rabbit, 134395, Cell Signaling, Massachusetts, USA), anti-HLTF (Rabbit, ab183042, Abcam Cambridge, UK), anti-ZRANB3 (Rabbit, 23111-1-AP, Proteintech, Manchester, UK), anti-β actin antibody (mouse, sc-47778, Santa Cruz Biotechnologies, Dallas, Texas, USA), anti-alpha Tubulin antibody (mouse, ab7291, Abcam Cambridge, UK). Chemiluminescence detection and quantification of protein levels were carried out in the linear range using ImageLab software on a ChemiDocMP System (BioRad Laboratories, München, Germany). Values for the protein of interest were corrected with values of the loading control.

Western blotting for expression analysis without immunoprecipitation followed the protocols described in [19]. Conditions of protein expression were based on preceding titration experiments to ensure comparable p53 levels.

### Cell cycle and apoptosis analysis

Cell cycle distribution and apoptosis in K562 cells were analyzed as described in [19].

### Transfection

Plasmids were transiently introduced in K562 and K562(*HR-EGFP/3*′*EGFP*) via electroporation (GenePulser Xcell, BioRad Laboratories, München, Germany) as described in [19]. In Saos-2 and U2OS cells plasmids/siRNAs were transiently introduced using Amaxa Cell Line Nucleofector Solution V and Amaxa device: program D-24 for Saos-2, X-01 for U2OS (Lonza, Basel Switzerland) [20]. Introduction of siRNAs into HSPC cells relied on Amaxa B cell Nucleofector Solution and Amaxa device program U-08.

### Plasmids and siRNAs

The vectors pcDNA-p53α, pcDNA-p53β, pcDNA-p53γ, pcDNA-Δ40p53α, pcDNA-Δ133p53α, and pcDNA-Δ160p53α used in this study were generated at the University of Dundee as described in ([16, 26]). pGEX-4T MDM2 WT was a gift from Mien-Chie Hung (Addgene plasmid #16237; http://n2t.net/addgene:16237; RRID:Addgene_16237) [69]. pcDNA3-HA p21 was a gift from Jaewhan Song (Addgene plasmid #78782; http://n2t.net/addgene:78782; RRID:Addgene_78782) [70]. For knockdown experiments of POLι, HLTF, and ZRANB3 we engaged two shRNA-expressing plasmids each, which were previously established in [19]. To silence all p53-isoforms, the following siRNAs were used: GACUCCAGUGGUAAUCUAC and GGAGAAUAUUUCACCCUUC, to target Δ133/Δ160p53: GGAGGUGCUUACGCAUGUU and CUUGUGCCCUGACUUUCAA.

### Statistics

Graphic presentation of data, statistical analysis, calculation of mean values, standard deviation, and standard error of the mean were performed with GraphPadPrism8.4 software (San Diego, CA). For calculation of statistically significant differences in recombination measurements, the Kruskal−Wallis test followed by Mann−Whitney two-tailed test was used. For calculation of statistically significant differences in DNA fiber spreading analysis and immunofluorescence experiments Dunns-multiple comparison test was used. For calculation of statistically significant differences in Western Blot analysis, ANOVA followed by paired t-test or Friedmann test followed by Wilcoxon-matched pair signed-rank test was used.

## Supplementary information

Supplementary Figures S1−S8

Extended material

## Data Availability

The datasets generated during and/or analyzed during the current study are available from the corresponding authors upon request.

## References

[1] Eliyahu D, Michalovitz D, Eliyahu S, Pinhasi-Kimhi O, Oren M (1989). Wild-type p53 can inhibit oncogene-mediated focus formation. Proc Natl Acad Sci USA.

[2] Finlay CA, Hinds PW, Levine AJ (1989). The p53 proto-oncogene can act as a suppressor of transformation. Cell.

[3] Lane DP (1992). p53, guardian of the genome. Nature.

[4] Bargonetti J, Friedman PN, Kern SE, Vogelstein B, Prives C (1991). Wild-type but not mutant p53 immunopurified proteins bind to sequences adjacent to the SV40 origin of replication. Cell.

[5] Kern SE, Kinzler KW, Bruskin A, Jarosz D, Friedman P, Prives C (1991). Identification of p53 as a sequence-specific DNA-binding protein. Science.

[6] Yonish-Rouach E, Resnftzky D, Lotem J, Sachs L, Kimchi A, Oren M (1991). Wild-type p53 induces apoptosis of myeloid leukaemic cells that is inhibited by interleukin-6. Nature.

[7] El-Deiry WS, Kern SE, Pietenpol JA, Kinzler KW, Vogelstein B (1992). Definition of a consensus binding site for p53. Nat Genet.

[8] Dutta A, Ruppert JM, Aster JC, Winchester E (1993). Inhibition of DNA replication factor RPA by p53. Nature.

[9] Li R, Botchan MR (1993). The acidic transcriptional activation domains of VP16 and p53 bind the cellular replication protein A and stimulate in vitro BPV-1 DNA replication. Cell.

[10] Lee S, Elenbaas B, Levine A, Griffith J (1995). p53 and its 14 kDa C-terminal domain recognize primary DNA damage in the form of insertion/deletion mismatches. Cell.

[11] Mummenbrauer T, Janus F, Müller B, Wiesmüller L, Deppert W, Grosse F (1996). p53 Protein exhibits 3′-to-5′ exonuclease activity. Cell.

[12] Stürzbecher HW, Donzelmann B, Henning W, Knippschild U, Buchhop S (1996). p53 is linked directly to homologous recombination processes via RAD51/RecA protein interaction. EMBO J.

[13] Dudenhöffer C, Rohaly G, Will K, Deppert W, Wiesmüller L (1998). Specific mismatch recognition in heteroduplex intermediates by p53 suggests a role in fidelity control of homologous recombination. Mol Cell Biol.

[14] Offer H, Wolkowicz R, Matas D, Blumenstein S, Livneh Z, Rotter V (1999). Direct involvement of p53 in the base excision repair pathway of the DNA repair machinery. FEBS Lett.

[15] Vieler M, Sanyal S (2018). p53 isoforms and their implications in cancer. Cancers.

[16] Bourdon J, Fernandes K, Murray-zmijewski F, Liu G, Diot A, Xirodimas DP (2005). p53 isoforms can regulate p53 transcriptional activity. Genes Dev.

[17] Baugh EH, Ke H, Levine AJ, Bonneau RA, Chan CS (2018). Why are there hotspot mutations in the TP53 gene in human cancers?. Cell Death Differ.

[18] Khoury MP, Bourdon JC (2011). P53 isoforms: an intracellular microprocessor?. Genes Cancer.

[19] Hampp S, Kiessling T, Buechle K, Mansilla SF, Thomale J, Rall M (2016). DNA damage tolerance pathway involving DNA polymerase ι and the tumor suppressor p53 regulates DNA replication fork progression. Proc Natl Acad Sci USA.

[20] Biber S, Pospiech H, Gottifredi V, Wiesm L (2020). Multiple biochemical properties of the p53 molecule contribute to activation of polymerase iota-dependent DNA damage tolerance. Nucleic Acids Res.

[21] Ireno IC, Wiehe RS, Stahl AI, Hampp S, Aydin S, A.Troester M (2014). Modulation of the poly (ADP-ribose) polymerase inhibitor response and DNA recombination in breast cancer cells by drugs affecting endogenous wild-type p53. Carcinogenesis.

[22] Ihle M, Biber S, Schroeder IS, Blattner C, Deniz M, Damia G (2021). Impact of the interplay between stemness features, p53 and pol iota on replication pathway choices. Nucleic Acids Res.

[23] Joruiz SM, Bourdon JC (2016). p53 isoforms: key regulators of the cell fate decision. Cold Spring Harb Perspect Med.

[24] Akyüz N, Boehden GS, Süsse S, Rimek A, Preuss U, Scheidtmann K-H (2002). DNA substrate dependence of p53-mediated regulation of double-strand break repair. Mol Cell Biol.

[25] Restle A, Färber M, Baumann C, Böhringer M, Scheidtmann KH, Müller-Tidow C (2008). Dissecting the role of p53 phosphorylation in homologous recombination provides new clues for gain-of-function mutants. Nucleic Acids Res.

[26] Marcel V, Perrier S, Aoubala M, Ageorges S, Groves MJ, Diot A (2010). Δ160p53 is a novel N-terminal p53 isoform encoded by Δ133p53 transcript. FEBS Lett.

[27] Técher H, Koundrioukoff S, Azar D, Wilhelm T, Carignon S, Brison O (2013). Replication dynamics: biases and robustness of DNA fiber analysis. J Mol Biol.

[28] Livneh Z (2006). Keeping mammalian mutation load in check: regulation of the activity of error-prone DNA polymerases by p53 and p21. Cell Cycle.

[29] Klusmann I, Rodewald S, Müller L, Friedrich M, Wienken M, Li Y (2016). p53 activity results in DNA replication fork processivity. Cell Rep.

[30] Klusmann I, Wohlberedt K, Magerhans A, Teloni F, Korbel JO, Altmeyer M (2018). Chromatin modifiers Mdm2 and RNF2 prevent RNA:DNA hybrids that impair DNA replication. Proc Natl Acad Sci USA.

[31] Fujita K, Mondal AM, Horikawa I, Nguyen GH, Kumamoto K, Sohn JJ (2009). p53 isoforms Delta133p53 and p53beta are endogenous regulators of replicative cellular senescence. Nat Cell Biol.

[32] Hafsi H, Santos-Silva D, Courtois-Cox S, Hainaut P (2013). Effects of Δ40p53, an isoform of p53 lacking the N-terminus, on transactivation capacity of the tumor suppressor protein p53. BMC Cancer.

[33] von Muhlinen N, Horikawa I, Alam F, Isogaya K, Lissa D, Vojtesek B (2018). p53 isoforms regulate premature aging in human cells. Oncogene.

[34] Tomasz M (1995). Mitomycin C: small, fast, and deadly (but very selective). Chem Biol.

[35] Amunugama R, Willcox S, Wu RA, Abdullah UB, El-Sagheer AH, Brown T (2018). Replication fork reversal during DNA interstrand crosslink repair requires CMG unloading. Cell Rep.

[36] Langevin F, Crossan GP, Rosado IV, Arends MJ, Patel KJ (2011). Fancd2 counteracts the toxic effects of naturally produced aldehydes in mice. Nature.

[37] Gobert C, Skladanowski A, Larsen AK (1999). The interaction between p53 and DNA topoisomerase I is regulated differently in cells with wild-type and mutant p53. Proc Natl Acad Sci USA.

[38] Dudenhöffer C, Kurth M, Janus F, Deppert W, Wiesmüller L (1999). Dissociation of the recombination control and the sequence-specific transactivation function of P53. Oncogene.

[39] Lei J, Qi R, Tang Y, Wang W, Wei G, Nussinov R (2019). Conformational stability and dynamics of the cancer-associated isoform Δ133p53β are modulated by p53 peptides and p53-specific DNA. FASEB J.

[40] Pilzecker B, Buoninfante OA, Jacobs H (2019). DNA damage tolerance in stem cells, ageing, mutagenesis, disease and cancer therapy. Nucleic Acids Res.

[41] Banks D, Wu M, Higa LA, Gavrilova N, Quan J, Ye T (2006). L2DTL/CDT2 and PCNA interact with p53 and regulate p53 polyubiquitination and protein stability through MDM2 and CUL4A/DDB1 complexes. Cell Cycle.

[42] Lin J, Chen J, Elenbaas B, Levine AJ (1994). Several hydrophobic amino acids in the p53 amino-terminal domain are required for transcriptional activation, binding to mdm-2 and the adenovirus 5 E1B 55-kD protein. Genes Dev.

[43] Fischer M (2017). Census and evaluation of p53 target genes. Oncogene.

[44] el-Deiry WS, Tokino T, Velculescu VE, Levy DB, Parsons R, Trent JM (1993). WAF1, a potential mediator of p53 tumor suppression. Cell.

[45] Barak Y, Juven T, Haffner R, Oren M (1993). mdm2 expression is induced by wild type p53 activity. EMBO J.

[46] Mansilla SF, de la Vega MB, Calzetta NL, Siri SO, Gottifredi V (2020). Cdk-independent and pcna-dependent functions of p21 in dna replication. Genes.

[47] Ahn J, Poyurovsky MV, Baptiste N, Beckerman R, Cain C, Mattia M (2009). Dissection of the sequence-specific DNA binding and exonuclease activities reveals a superactive yet apoptotically impaired mutant p53 protein. Cell Cycle.

[48] Romanova LY, Willers H, Blagosklonny MV, Powell SN (2004). The interaction of p53 with replication protein A mediates suppression of homologous recombination. Oncogene.

[49] Jayaraman J, Prives C (1995). Activation of p53 sequence-specific DNA binding by short single strands of DNA requires the p53 C-terminus. Cell.

[50] Larsen AK, Gobert C (1999). DNA topoisomerase I in oncology: Dr Jekyll or Mr Hyde?. Pathol Oncol Res.

[51] Fischbach A, Krüger A, Hampp S, Assmann G, Rank L, Hufnagel M (2018). The C-terminal domain of p53 orchestrates the interplay between non-covalent and covalent poly(ADP-ribosyl)ation of p53 by PARP1. Nucleic Acids Res.

[52] Serrano MA, Li Z, Dangeti M, Musich PR, Patrick S, Roginskaya M (2013). DNA-PK, ATM, and ATR collaboratively regulate p53-RPA interaction to facilitate homologous recombination DNA repair. Oncogene.

[53] Schlacher K, Wu H, Jasin M (2012). A distinct replication fork protection pathway connects Fanconi anemia tumor suppressors to RAD51-BRCA1/2. Cancer Cell.

[54] Romanova LY, Mushinski F, Kovalchuk AL (2018). Transcriptional activation of p21Waf1 contributes to suppression of HR by p53 in response to replication arrest induced by camptothecin. Oncotarget.

[55] Xu X, Vaithiyalingam S, Glick GG, Mordes DA, Chazin WJ, Cortez D (2008). The basic cleft of RPA70N binds multiple checkpoint proteins, including RAD9, to regulate ATR signaling. Mol Cell Biol.

[56] Ano Bom APD, Rangel LP, Costa DCF, de Oliveira GAP, Sanches D, Braga CA (2012). Mutant p53 aggregates into prion-like amyloid oligomers and fibrils: implications for cancer. J Biol Chem.

[57] Silva JL, Cino EA, Soares IN, Ferreira VF, AP de Oliveira G (2018). Targeting the prion-like aggregation of mutant p53 to combat cancer. Acc Chem Res.

[58] Ghosh S, Ghosh D, Ranganathan S, Anoop A, P SK, Jha NN (2014). Investigating the intrinsic aggregation potential of evolutionarily conserved segments in p53. Biochemistry.

[59] Li R, Sutphin PD, Schwartz D, Matas D, Almog N, Wolkowicz R (1998). Mutant p53 protein expression interferes with p53-independent apoptotic pathways. Oncogene.

[60] Bargonetti J, Prives C (2019). Gain-of-function mutant p53: history and speculation. J Mol Cell Biol.

[61] Krois AS, Dyson HJ, Wright PE (2018). Long-range regulation of p53 DNA binding by its intrinsically disordered N-terminal transactivation domain. Proc Natl Acad Sci USA.

[62] Tu Q, Gong H, Yuan C, Liu G, Huang J, Li Z (2020). Δ133p53/FLp53 predicts poor clinical outcome in esophageal squamous cell carcinoma. Cancer Manag Res.

[63] Nutthasirikul N, Limpaiboon T, Leelayuwat C, Patrakitkomjorn S, Jearanaikoon P (2013). Ratio disruption of the Δ133p53 and TAp53 isoform equilibrium correlates with poor clinical outcome in intrahepatic cholangiocarcinoma. Int J Oncol.

[64] Kazantseva M, Mehta S, Eiholzer RA, Gimenez G, Bowie S, Campbell H (2019). The Δ133p53β isoform promotes an immunosuppressive environment leading to aggressive prostate cancer. Cell Death Dis.

[65] Bai G, Kermi C, Stoy H, Schiltz CJ, Bacal J, Zaino AM (2020). HLTF promotes fork reversal, limiting replication stress resistance and preventing multiple mechanisms of unrestrained DNA synthesis. Mol Cell.

[66] Nayak S, Calvo JA, Cantor SB (2021). Targeting translesion synthesis (TLS) to expose replication gaps, a unique cancer vulnerability. Expert Opin Ther Targets.

[67] Kraft D, Rall M, Volcic M, Metzler E, Groo A, Stahl A (2015). NF-κB-dependent DNA damage-signaling differentially regulates DNA double-strand break repair mechanisms in immature and mature human hematopoietic cells. Leukemia.

[68] Schindelin J, Arganda-Carreras I, Frise E, Kaynig V, Longair M, Pietzsch T (2012). Fiji: an open-source platform for biological-image analysis. Nat Methods.

[69] Zhou BP, Liao Y, Xia W, Zou Y, Spohn B, Hung MC (2001). HER-2/neu induces p53 ubiquitination via Akt-mediated MDM2 phosphorylation. Nat Cell Biol.

[70] Lee M-S, Seo J, Choi DY, Lee E-W, Ko A, Ha N-C (2013). Stabilization of p21 (Cip1/WAF1) following Tip60-dependent acetylation is required for p21-mediated DNA damage response. Cell Death Differ.

