# Supplementary material for: p53 isoforms differentially impact on the POLι dependent DNA damage tolerance pathway
Source: Cell Death Dis. 2021 Oct 13;12(10):941. doi: 10.1038/s41419-021-04224-3 (PMC8514551; doi:10.1038/s41419-021-04224-3)
Supplement: Supplementary file 1 — Supplementary Figures S1−S8 [file 41419_2021_4224_MOESM1_ESM.pdf]

Supplementary Fig. S1

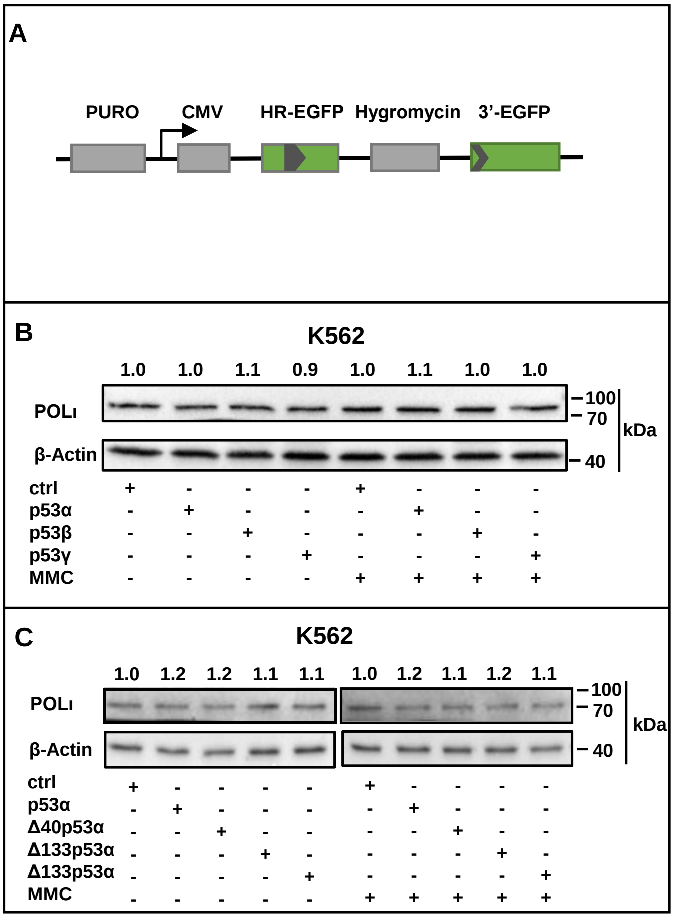

Supplementary Fig. S1: Principle of the analysis of replication-associated recombination and expression of POLι in K562.

A: **Schematic presentation of the EGFP-based recombination substrate (*HR-EGFP/3`-EGFP*).** K562(*HR-EGFP/3`-EGFP*) cells with chromosomally integrated substrate were used to determine the recombination (rec.) fold changes [24]. Hygromycin=hygromycin resistance cassette; PURO=puromycin resistance cassette. The kinked arrow indicates the promoter, the grey, fat arrow a frameshift mutation within the chromophore coding region resulting in an inactive *HR-EGFP* mutant, the grey, thin arrow replacement of the *EGFP* start codon by two stop codons generating an inactive *3`-EGFP* mutant.

B,C: POLι expression levels. K562 cells were transfected with expression plasmids of (**B**) p53α, p53β, p53γ or empty vector (ctrl) and (**C**) p53α, Δ40p53α, Δ133p53α, Δ160p53α or empty vector (ctrl). 48 h post-transfection, cells were either mock- or MMC-treated (3 µM, 45 min, 3 h release), proteins were harvested and immunoblotting was performed to investigate POLι expression levels. β-Actin served as loading control. Quantification of POLι was carried out with ImageLab software and normalized to values of β-Actin. Mean values (≥ 3 experiments) are shown above the representative western blot images.

Supplementary Fig. S2

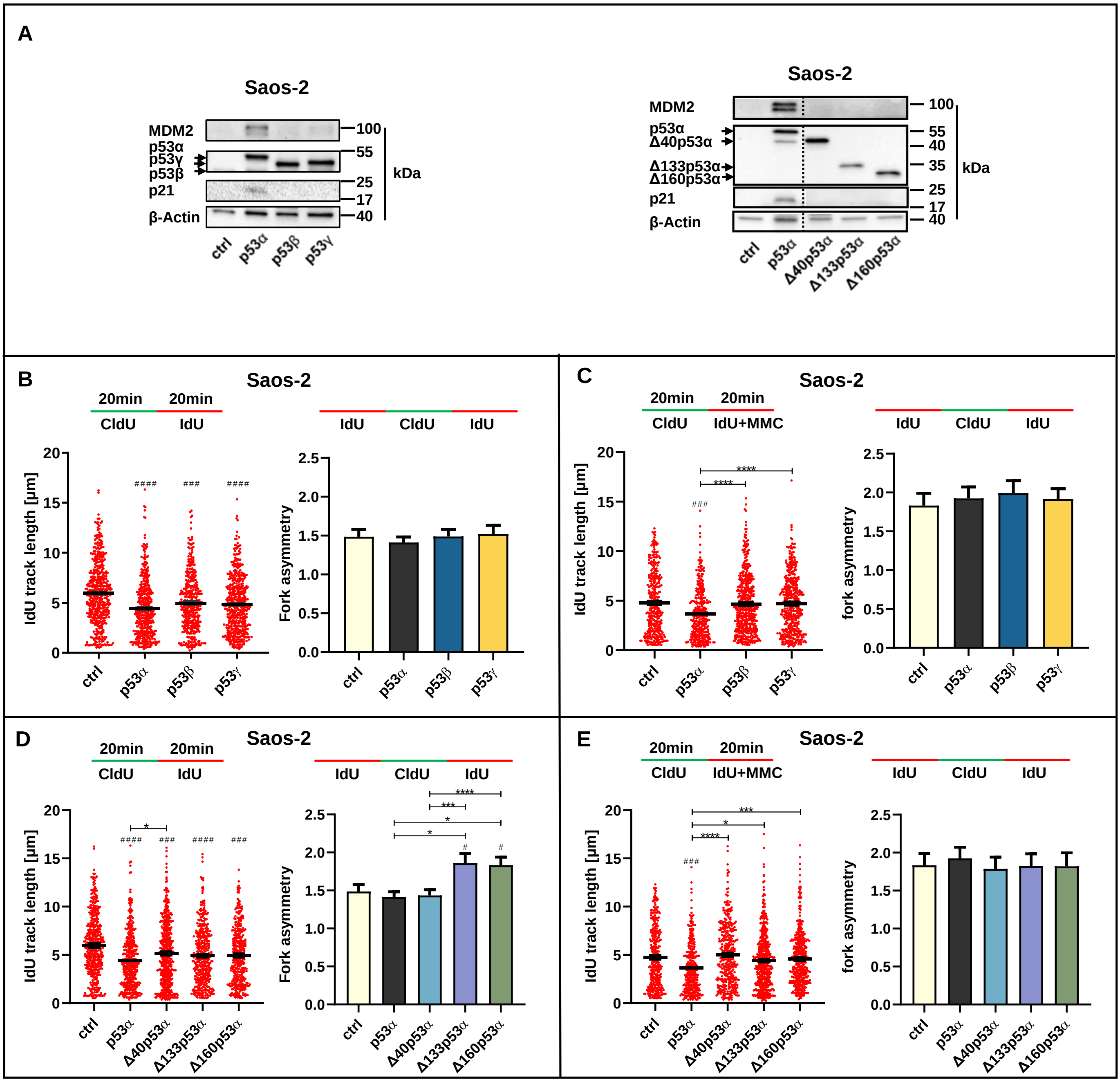

Supplementary Fig. S2: Analysis of protein expression and replication dynamics in Saos-2 cells.

A: Expression of p53 isoforms and transcriptional targets MDM2 and p21 in Saos-2 cells. Saos-2 cells were transfected with empty vector or expression plasmids for p53α, p53β and p53γ (left panel). 24 h post-transfection, proteins were harvested and processed for immunoblotting to visualize expression levels of MDM2, p53 and p21 in Saos-2 cells. β-Actin served as loading control. Saos-2 cells were transfected with empty vector or expression plasmids for p53α, Δ40p53α, Δ133p53α and Δ160p53α (right panel). 24 h post-transfection, proteins were harvested and processed for immunoblotting to visualize expression levels of MDM2, p53 and p21 in Saos-2 cells. β-Actin served as loading control. Image parts separated by stippled lines were derived from the same image of the same blot but were cropped to remove unrelated samples.

B-E: **Replication dynamics in Saos-2 cells.** Saos-2 cells were transfected with expression plasmids for (**B, C**) p53α, p53β, p53γ or empty vector (ctrl) or (**D, E**) p53α, Δ40p53α, Δ133p53α, Δ160p53α or empty vector (ctrl). 24 h post-transfection DNA fiber spreading assays were performed. Experimental design was as described in Fig. 2 and shown in the experimental outline in the upper panels of (**B-E**). Both CldU- and IdU-tracks of ongoing forks were measured but for clarity graphic presentations of track-length evaluations focus on IdU-tracks (≥325 to ≥358 fibers in two independent biological experiments). For FA analysis, ≥39 to ≥ 47 fibers in two independent biological experiments were measured. For graphic presentation, calculation of SEM and statistically significant differences via Dunn`s multiple comparison test GraphPadPrism8.4 software was used. # indicates a statistically significant difference between the empty vector and the respective p53-isoform.*(#)*P*<0.05, ***(# # #)*P*<0.001, ****P(# # # #) < 0.0001.

Supplementary Fig. S3

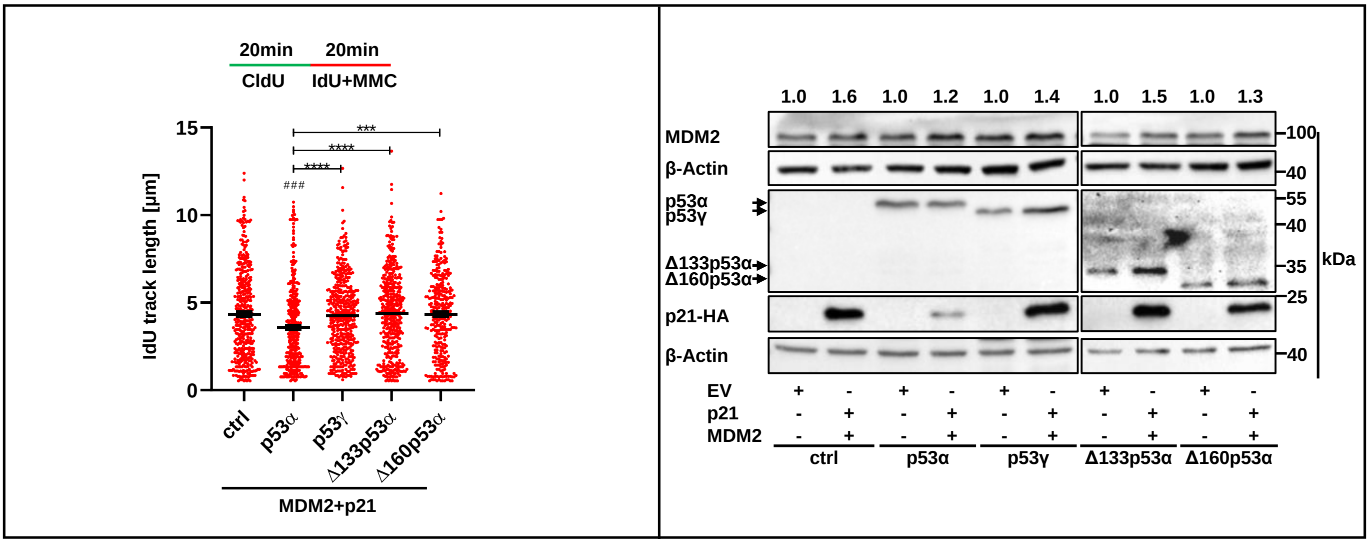

Supplementary Fig. S3: Replication dynamics after expression of exogenous MDM2 and p21.
K562 cells were co-transfected with expression plasmids for p53α, p53γ, Δ133p53α, Δ160p53α or control vector (ctrl) and the expression plasmids for MDM2, HA-tagged p21 or empty vector (EV). Proteins were harvested and DNA fiber spreading assay was performed 48 h post-transfection. Experimental design was as described in Fig. 2. Both CldU- and IdU-tracks of ongoing forks were measured but for clarity graphic presentation in the left panel focuses on IdU-tracks (≥314 fibers from two independent biological experiments). For graphic presentation, calculation of SEM and statistically significant differences via Dunn`s multiple comparison test GraphPadPrism8.4 software was used. # indicates a statistically significant difference between the control vector and the respective p53-isoform. .*(#)P<0.05, ***(# # #)P<0.001, ****P(# # # #) < 0.0001. Right panel shows protein expression levels of samples transfected and treated as the fiber assay samples shown in the left panel. β-Actin served as loading control. Quantification of MDM2 was carried out with ImageLab software and normalized to values of β-Actin. One representative immunoblot (out of 2) and the corresponding quantification is shown.

Supplementary Fig. S4

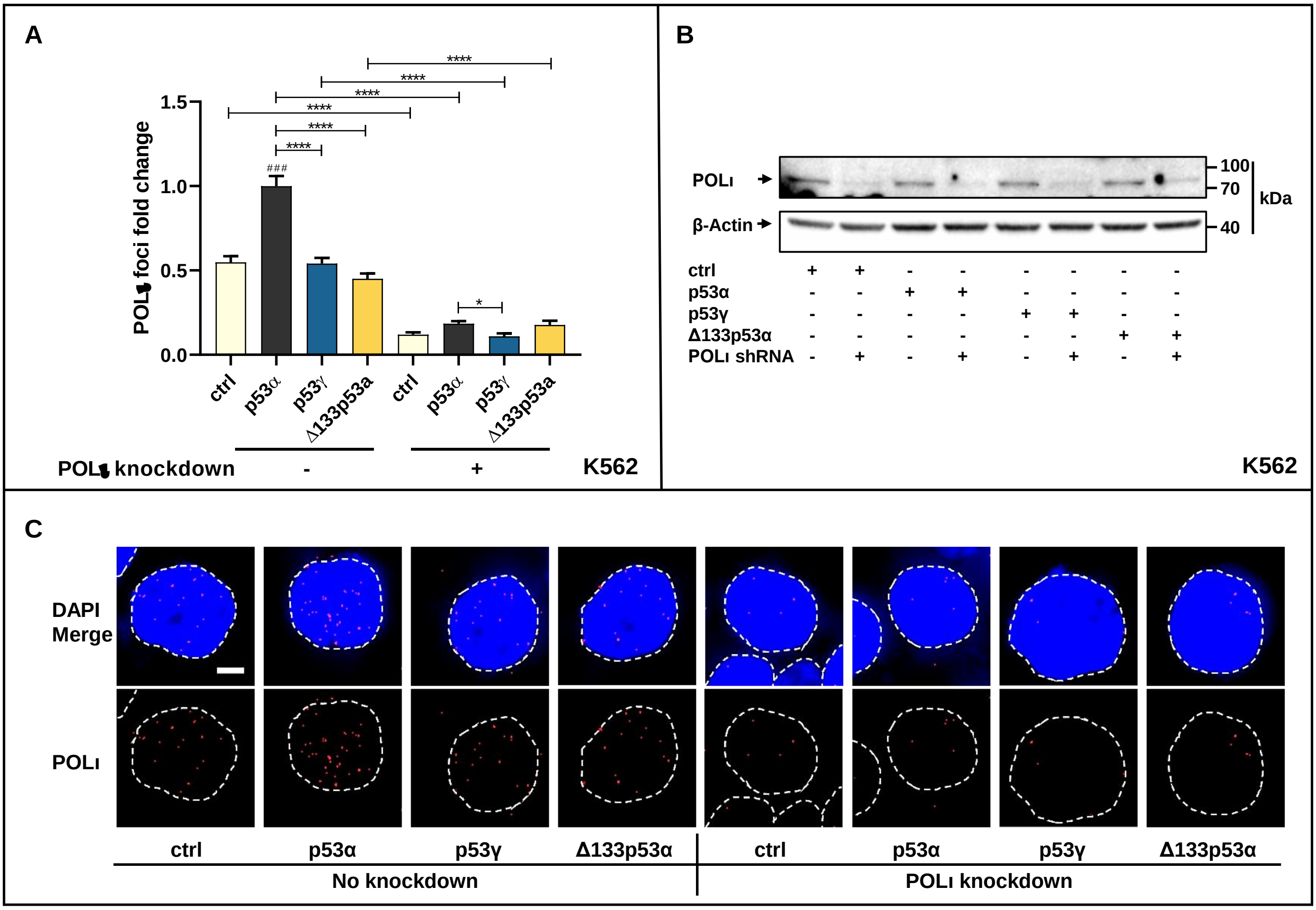

Supplementary Fig. S4: Immunofluorescence microscopy of POLı in K562 cells expressing p53 isoforms with or without knockdown of POLı.

K562 cells were transfected with empty vector (ctrl) or expression plasmids for p53α, p53γ or Δ133p53α together with empty vector shpRS or POLı-specific shRNA. 48 h after transfection, Cells were treated with MMC (3µM for 45 min, 3 h release) and processed for immunostaining to visualize POLı foci. At least 100 nuclei were scored in two independent experiments. Mean values for p53α expressing cells without POLı shRNA transfection were set to 1. Representative Western Blots of POLı expression levels are shown in (**B**). Error bars indicate mean ± SEM. Statistically significant differences calculated via Dunn's multiple comparisons test. Representative images of microscopic samples are shown in (**C**). *P<0.05, # # # P<0.001, **** P< 0.0001. (Scale bar: 5µm).

Supplementary Fig. S5

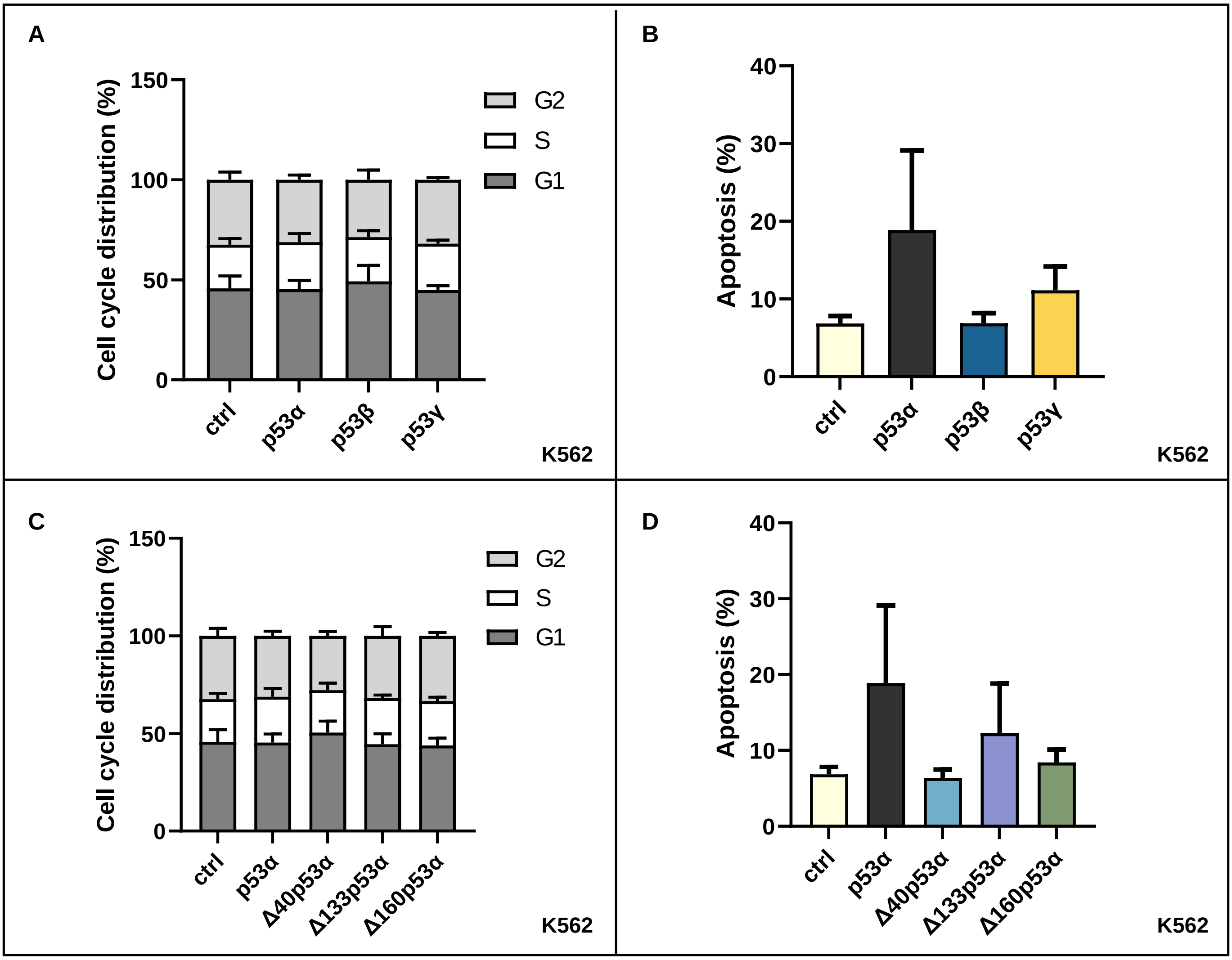

Supplementary Fig. S5: Cell cycle and apoptosis analysis of K562 cells expressing p53-isoforms after MMC treatment.

K562 cells were transfected with expression plasmids for p53α, alternative p53 isoforms [(**A**, **B**) p53β, p53γ or (**C**, **D**) Δ40p53α, Δ133p53α, Δ160p53α] or empty vector in controls (ctrl). Transfected cells were treated with MMC (3µM for 45 min). 48 h post-treatment, cells were harvested for fixation and staining by propodium iodide. Samples were then analyzed by flow cytometry to determine the cell cycle distribution (**A**, **C**) and the fraction of cells with a sub-G1 content indicating apoptosis (**B**, **D**). Viable cells were divided into G1 (dark gray), S (white), and G2 (light gray) phases in (**A**, **C**). The experiments shown in (**A**, **B**, **C, D**) were performed together, which is why values for ctrl and p53α are identical but separated in different panels for clarity. Error bars indicate mean ± SD from four measurements. Statistical analysis of cell cycle distribution and apoptosis was performed by paired Friedman test without revealing statistically significant differences.

Supplementary Fig. S6

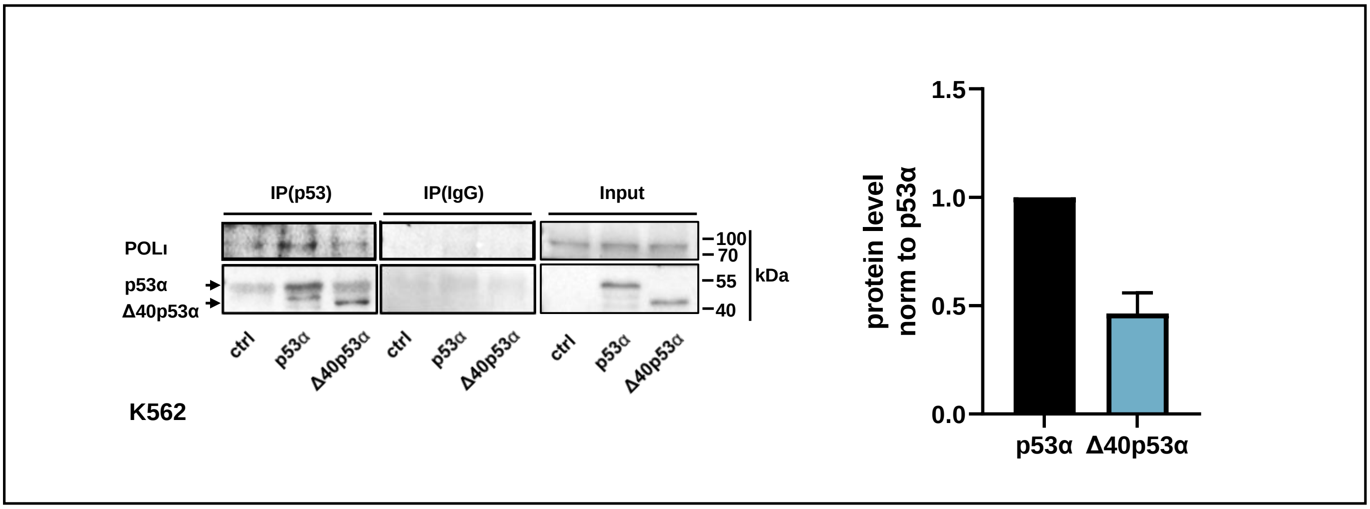

Supplementary Fig. S6: Δ40p53α is impaired in POLι binding.
K562 cells were transfected with expression plasmids for p53α, Δ40p53α or empty vector (ctrl). 48 h post-transfection, Co-immunoprecipitations were performed. Therefore, cells were lysed in 50 mM Tris, pH 8; 150 mM NaCl; 1 % NP40; complete protease inhibitor (Roche) cell lysis buffer. Pull-downs of p53 engaged a p53 antibody mix (mAb p53[Do11], MCA1704, BioRad and mAb p53[Pab421], OP03, Merck) or as control mouse-IgG (sc-2025, Santa Cruz). Subsequent immunoblotting was perfomed for the detection of POLι (anti-POLι, pAb, A310-314A, Bethyl) and p53 (anti-p53, mAb, p53[Do11], MCA1704, BioRad). Please note that light-chain specific peroxidase coupled secondary antibody was used. The left panel shows a representative Western Blot. The right panel shows the quantification of two immunoprecipitations. For the quantification of POLι ImageLab software was used. The level of POLι was corrected for values of the pull-down proteins p53α and Δ40p53α. The mean values of p53α were set to 1 each. Mean ±SD.

Supplementary Fig. S7

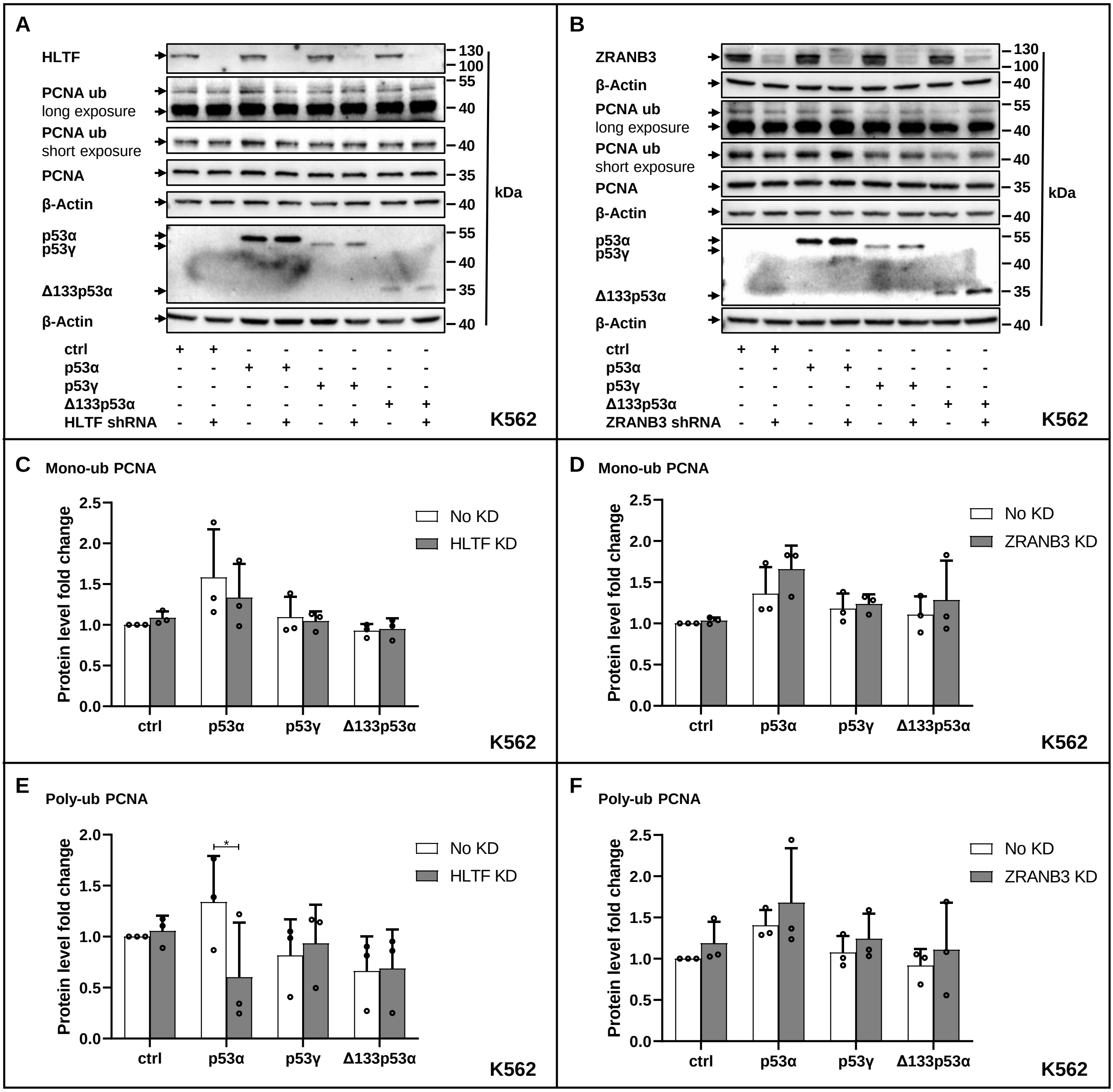

Supplementary Fig. S7: Knocking down HLTF in cells expressing p53α after MMC-treatment causes a decrease of PCNA-polyubiquitination.

K562 cells were transfected with shRNA empty vector or shRNA HLTF (**A**, **C**, **E**) / shRNA ZRANB3 (**B**, **D**, **F**) plasmids together with expression plasmids for p53α, alternative p53 isoforms (p53γ, Δ133p53α) or EV in controls (ctrl). 48 h after transfection, cells were MMC-treated (3 μM, 45 min, 3 h release) and lysed followed by immunoblotting using ubiquityl PCNA (Lys164, D5C7P , Cell Signaling, Massachusetts, USA) antibody, as well as antibodies against PCNA and p53. β-Actin was used as loading control. "ub" indicates ubiquitination. "KD" indicates knockdown. Quantification of respective protein expression level was carried out using ImageLab software. Levels of PCNA mono-/polyubiquitination were corrected for PCNA and normalized to ctrl (no KD) which was set to 1 on each blot. Error bars indicate mean ± SD. Statistically significant differences between No KD and either HLTF or ZRANB3 KD were calculated by paired t test. *P<0.05. **A**, **B**: Representative Western Blots from ctrl cells and cells expressing p53α or alternative isoforms without or with HLTF KD (**A**) / ZRANB3 KD (**B**). **C**, **D, E, F**: Quantification of PCNA mono-/polyubiquitination from 3 independent experiments.

Supplementary Fig. S8

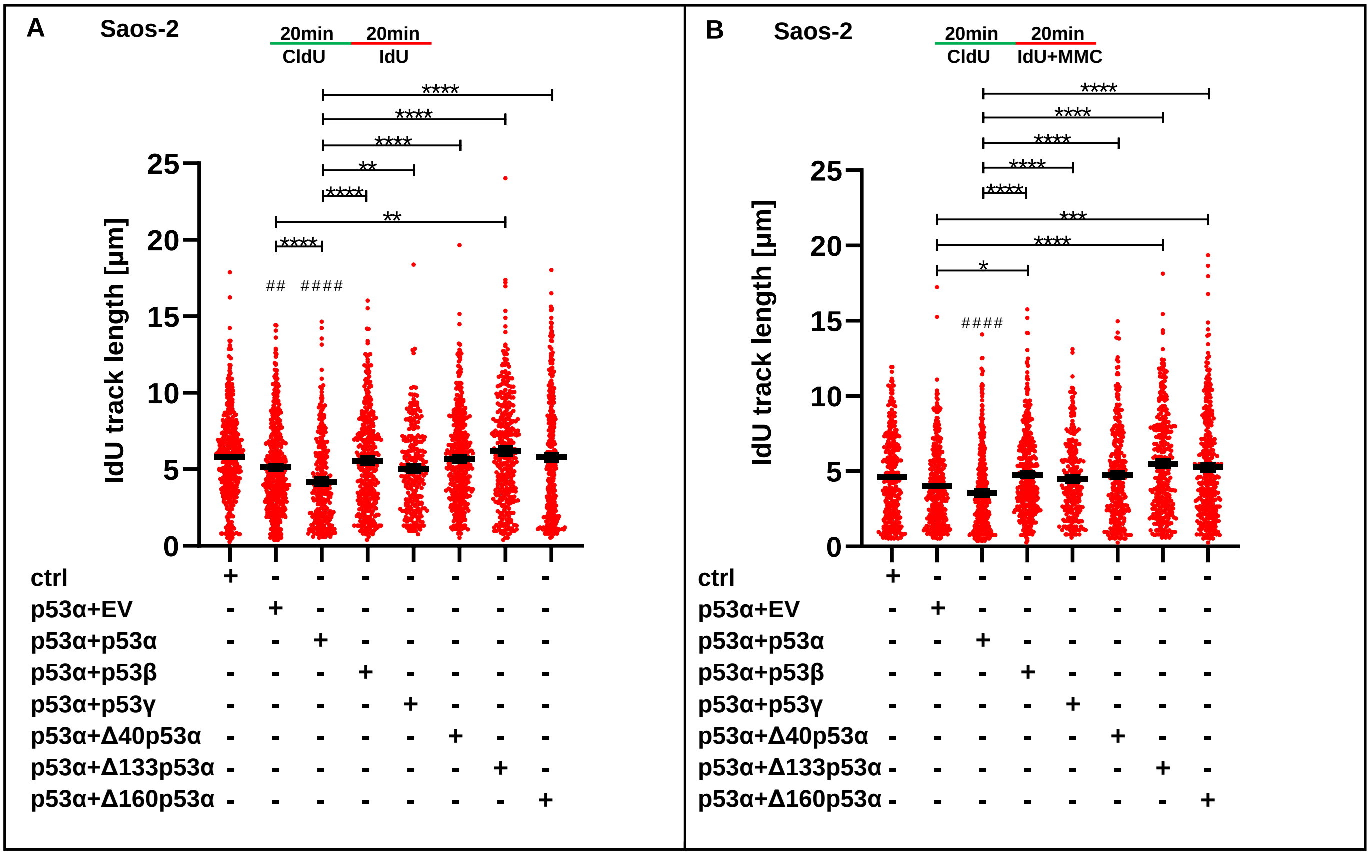

Supplementary Fig. S8: Replication dynamics after co-expression of p53α and alternative p53 isoforms in Saos-2 cells.

Saos-2 cells were transfected with a total amount of 20 µg plasmid DNA, containing either empty vector (EV) in controls (ctrl) or expression plasmid for p53α plus EV (p53α+EV) or for p53α plus p53α (p53α+p53α) or for p53α plus one of the alternative isoforms (p53α+p53β, p53α+p53γ, p53α+Δ40p53α, p53α+Δ133p53α, p53α+Δ160p53α). 24 h after transfection, DNA fiber spreading assays were performed. Experimental design was as described in the legend of Fig. 2. For graphic presentation, calculation of SEM and statistically significant differences via Dunn`s multiple comparison test GraphPadPrism8.4 software was used. # indicates a statistically significant difference between ctrl and the respective p53-isoform values.*(#)P<0.05, **(# #)P<0.01, ***(# # #)P<0.001, ****P(# # # #) < 0.0001. Both CldU- and IdU-tracks of ongoing forks were measured but for clarity graphic presentations focus on IdU-tracks (≥271 [**A, mock**] to ≥288 [**B, MMC**] fibers from two independent biological experiments).
