# Supplementary material for: p53 isoforms differentially impact on the POLι dependent DNA damage tolerance pathway
Source: Cell Death Dis. 2021 Oct 13;12(10):941. doi: 10.1038/s41419-021-04224-3 (PMC8514551; doi:10.1038/s41419-021-04224-3)
Supplement: Supplementary file 2 — Extended material [file 41419_2021_4224_MOESM2_ESM.pdf]

Extended Figure E1

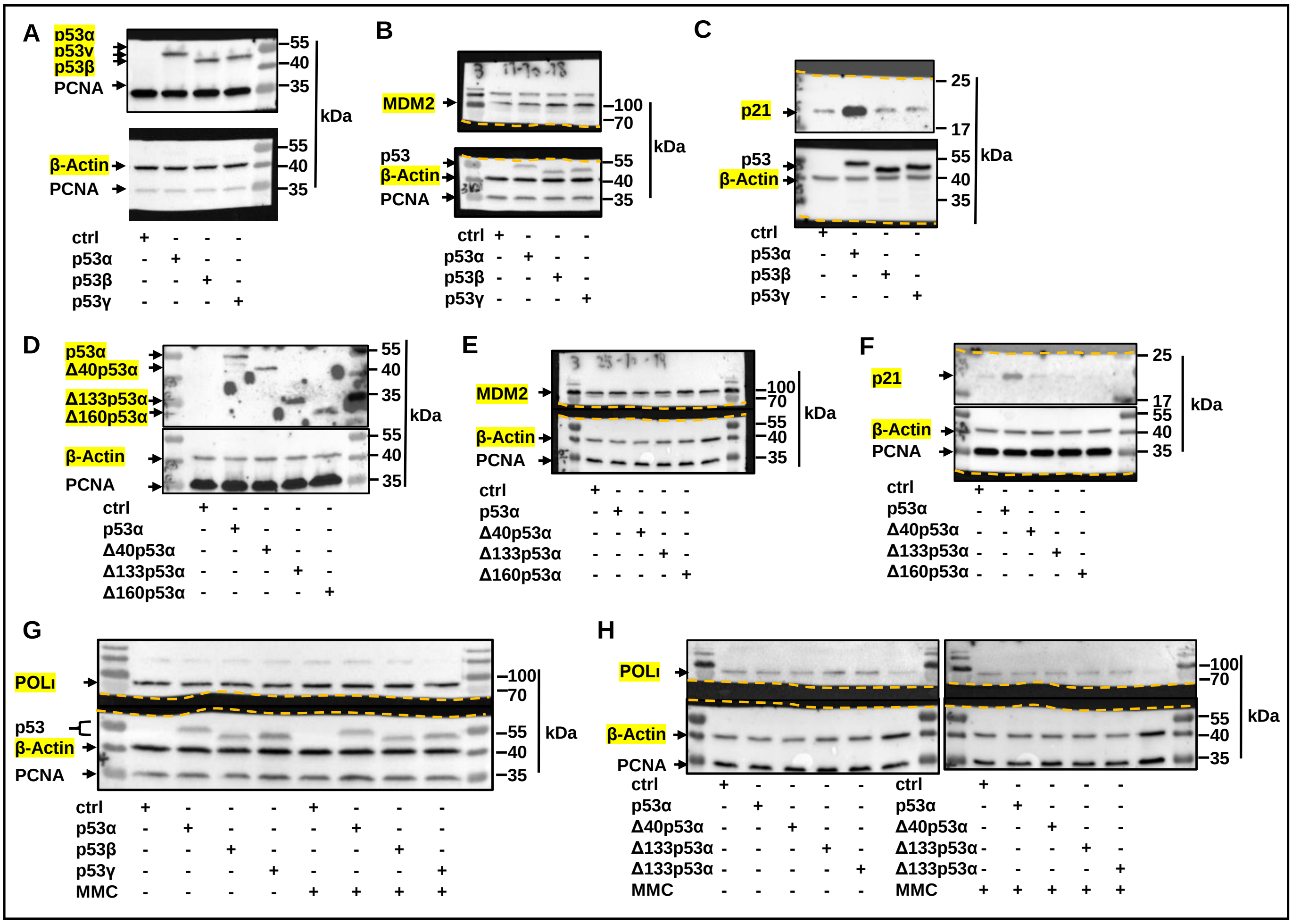

Fig E1: Original western blots of Fig 1/S1, bands shown in Fig 1/S1 are highlighted with yellow background. Yellow dashed lines on blots mark edges where blots were cut.

A, B, C: Original western blots of **Fig 1B** right panel.

D, E, F: Original western blots of **Fig 1C** right panel.

G, H: Original western blots of **Fig S1B and Fig S1C**.

Extended Figure E2

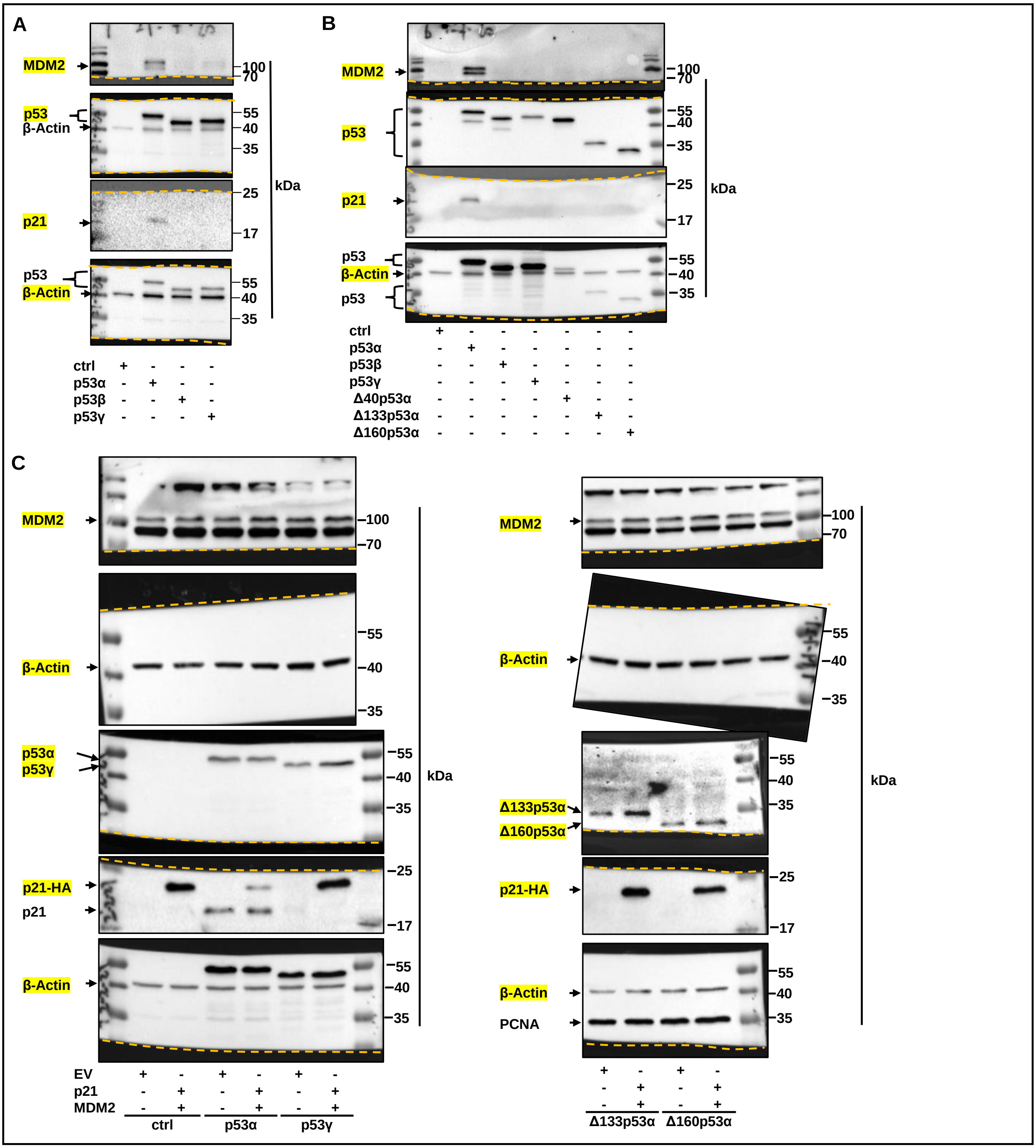

Fig E2: Original western blots of Fig S2/S3, bands shown in Fig S2/S3 are highlighted with yellow background. Yellow dashed lines on blots mark edges where blots were cut.

A, B: Original western blots of **Fig S2A**.

C: Original western blots of **Fig S3**.

Extended Figure E3

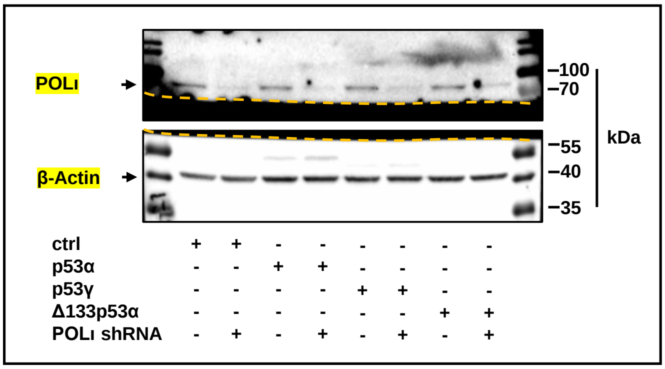

Fig E3: Original western blots of Fig S4B, bands shown in Fig S4B are highlighted with yellow background. Yellow dashed lines on blots mark edges where blots were cut.

Extended Figure E4

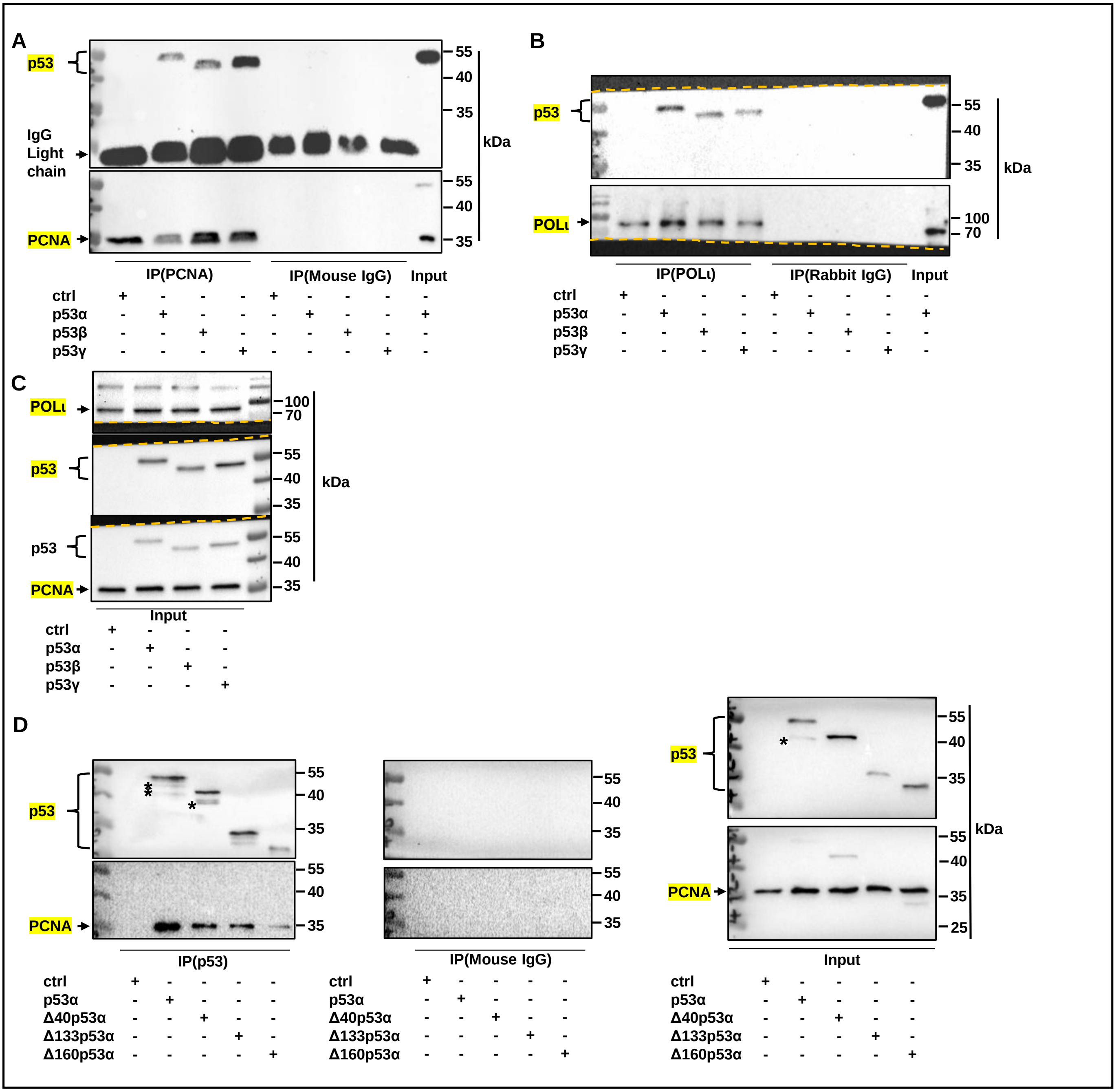

Fig E4: Original western blots of Fig 4, bands shown in Fig 4 are highlighted with yellow background. Yellow dashed lines on blots mark edges where blots were cut.

A, B, C: Original western blots of **Fig 4A**.

D: Original western blots of **Fig 4B**. Asterisks on blots indicate cleaved p53.

Extended Figure E5

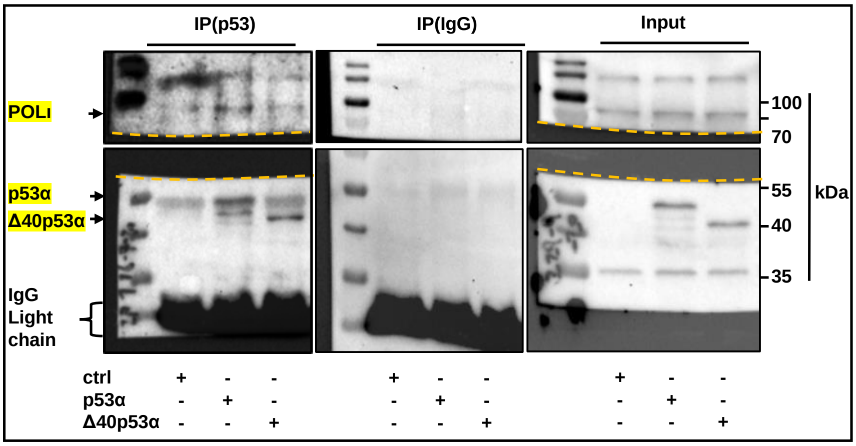

Fig E5: Original western blots of Fig S6, bands shown in Fig S6 are highlighted with yellow background. Yellow dashed lines on blots mark edges where blots were cut.

Extended Figure E6

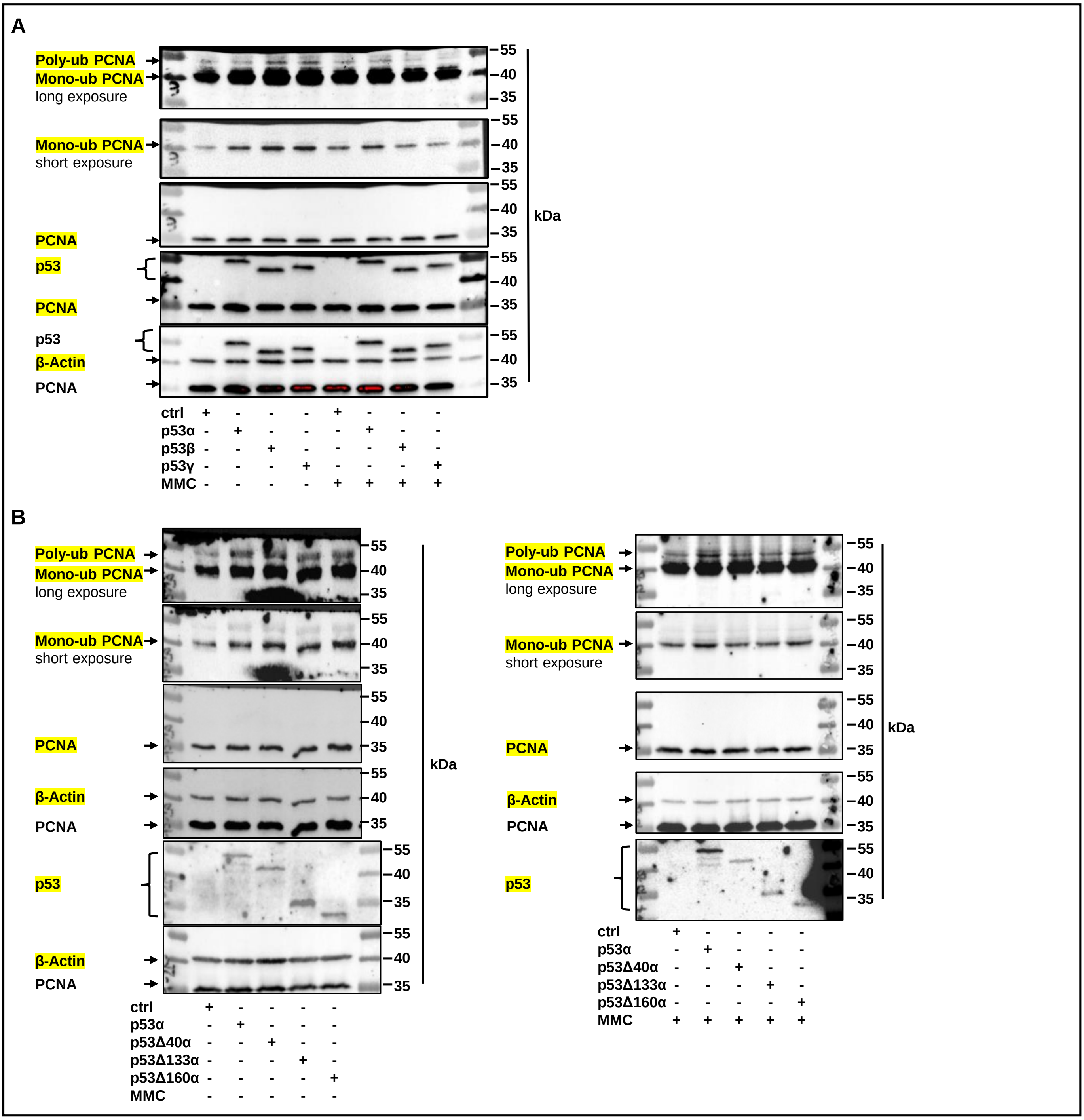

Fig E6: Original western blots of Fig 5, bands shown in Fig 5 are highlighted with yellow background.
A: Original western blots of **Fig 5A**.
B: Original western blots of **Fig 5B**.

Extended Figure E7

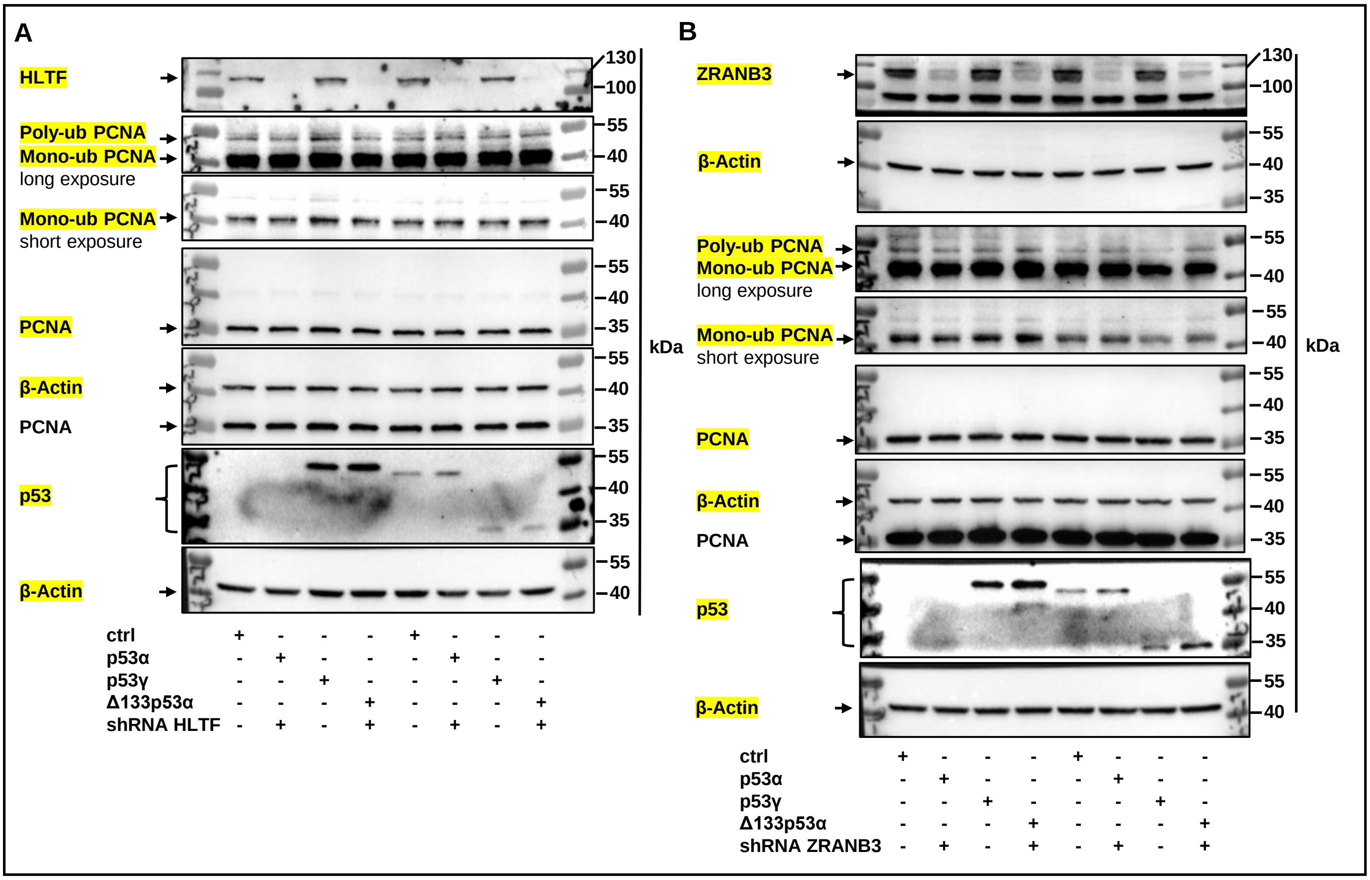

Fig E7: Original western blots of Fig S7, bands shown in Fig S7 are highlighted with yellow background.

A: Original western blots of **Fig S7A**.

B: Original western blots of **Fig S7B**.

Extended Figure E8

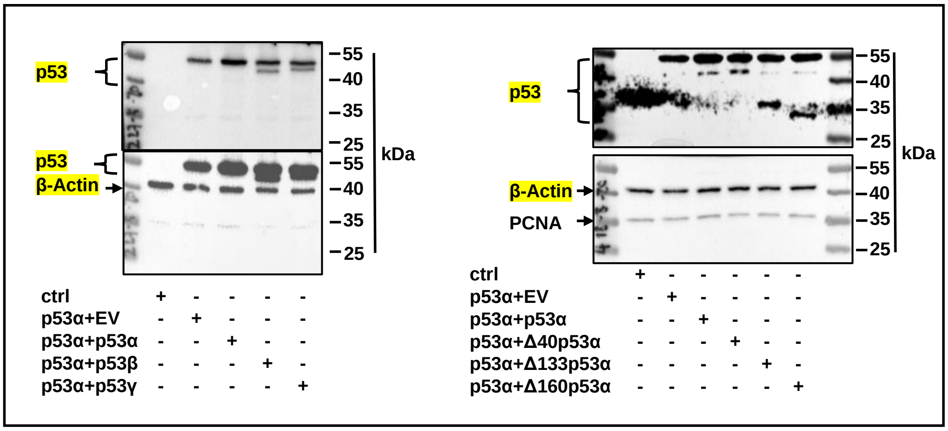

Fig E8: Original western blots of Fig 6F, bands shown in Fig 6F are highlighted with yellow background.

Extended Figure E9

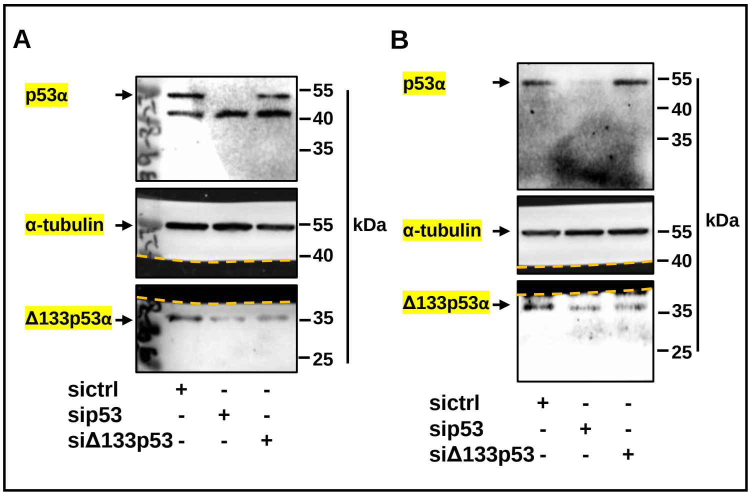

Fig E9: Original western blots of Fig 7, bands shown in Fig 7 are highlighted with yellow background. Yellow dashed lines on blots mark edges where blots were cut.

A: Original western blots of **Fig 7A**.

B: Original western blots of **Fig 7B**.
